# Diversity of Rock-Inhabiting Fungi in Tarragona Province, Spain

**DOI:** 10.3390/jof10030170

**Published:** 2024-02-22

**Authors:** Angie Paola Sastoque, José Francisco Cano-Lira, Alberto Miguel Stchigel

**Affiliations:** Mycology Unit, Medical School, Universitat Rovira i Virgili, C/Sant Llorenç 21, 43201 Reus, Tarragona, Spain; angiepaola.sastoque@urv.cat (A.P.S.); albertomiguel.stchigel@urv.cat (A.M.S.)

**Keywords:** *Ascomycota*, building materials, dark discoloration, extremophiles fungi, opportunistic pathogens

## Abstract

Rock-inhabiting fungi (RIF) are usually extremely tolerant or extremophilic, as they can survive on natural and artificial rocks despite being exposed to stressful conditions. RIF have serious negative effects on the appearance and cohesion of rocky substrates, causing the alteration and decomposition of building materials, but also on human and animal health, as they can act as opportunistic pathogens. Their identification is therefore of great importance, especially in urban areas. In the present study, culturing techniques *for* isolating fungi, and a polyphasic taxonomic approach to their identification, were used to assess the diversity of micromycetes that darken the surfaces of buildings in various villages and cities in Tarragona Province (Spain). Sixty-four species of RIF belonging to forty-one genera were identified, including a new genus (*Coccodomyces*) and the following six new fungal species: *Coccodomyces pleiosporus*, *Exophiala caementiphila*, *Exophiala multiformis*, *Neocatenulostroma spinulosum*, *Neodevriesia longicatenispora*, and *Paradevriesia holothallica*. Thus, we have established that building materials are ecological niches where a high biodiversity of RIF can develop.

## 1. Introduction

Within the wide variety of stressful environments, rocky surfaces (whether natural or artificial) are extreme for most microorganisms because of the limited availability of nutrients, the wide variation in temperatures throughout the seasons, the extremely drying effect of wind, and the harmful effects of solar radiation on microbial life through the action of ultraviolet radiation. However, genetic damage also can be caused by the emission of alpha particles generated by uranium decay, elements present in certain types of rocks used in construction (such as granite), exposure to environmental pollution, etc., as well as combinations of these factors [1,2,3,4]. Despite the extreme environmental conditions, rock substrates are inhabited by a high diversity of microorganisms, mostly bacteria and fungi. In fact, the proliferation of fungi is the main cause of the blackening (dark discoloration) of the outdoor surfaces of buildings and monuments. Previous studies have focused on the characterization of rock-inhabiting microbial communities and the nature and physiology of fungi growing on natural or artificial rocky substrates [5,6,7,8,9,10,11,12,13]. The fungi inhabiting monuments and urban buildings made of marble, stone, or concrete have drawn attention because of their negative impact on the cohesion and aesthetics of the construction materials. They play an important role in biodeterioration and influence public health by increasing the risk of contracting opportunistic mycoses and fungal allergies [1,7,13,14,15,16,17,18,19,20,21,22,23]. The free-living, oligotrophic, dematiaceous, and non-lichenized microfungi reported in these studies have been grouped and named according to their natural habitat as rock-inhabiting fungi (RIF), although they have been isolated even from non-rocky surfaces of urban buildings and living plants, and have been reported as opportunistic animal or human pathogens [4,24,25,26,27]. RIF, as mentioned above, possess peculiar physiological and structural features related to stress tolerance, such as their oligotrophic metabolism, which allows them to survive in substrates with a limited amount and diversity of nutrients but with a wide fluctuation of available water. This would cause the fungi to assimilate a wide spectrum of nitrogen and carbon sources, including recalcitrant carbon sources and even aliphatic and aromatic hydrocarbons [24,28,29,30,31,32,33]. In addition, RIF have thick, highly-melanized cell walls, a feature that protects the fungal cell against the damaging effects of a wide range of electromagnetic radiation (i.e., non-ionizing UV, ionizing X-rays, ɣ- and ß-radiation), extreme temperatures, and osmotic shock, and allows for mechanical penetration into hard inorganic materials and tissues of host plants or animals, including humans [4,24,34,35,36].

RIF can be divided into groups according to their morphological traits. De Hoog and Hermanides-Nijhof [37] introduced the term “**meristematic fungi**” to describe slow-growing fungi that form cauliflower-like colonies and aggregates of thick-walled melanized cells. They are reproduced by isodiametric enlargement and subdividing cells, releasing propagules by disarticulation or by endogenous conidiogenesis. Sometimes meristematic fungi produce few blastic conidia and/or budding cells. The authors also introduced the term “**black yeasts**” to describe a group of fungi that have in common melanized cell walls and the production of daughter yeast-like cells by monopolar or multilateral budding, which may be embedded in an extracellular polymeric molecule’s matrix. Most of these fungi can also produce hyphae and conidia from phialides or anellides [26,37]. In 1982, the term “**microcolonial fungi**” (MCF) was introduced by Staley et al. [38] to refer to the appearance and in situ growth of fungal colonies that are cauliflower-like, fairly small (up to 1 mm diameter), spherical, smooth, brown or black, that can be found growing on rocky substrates, glass, or metal surfaces, and whose micromorphology is characterized by meristematic or yeast-like growth with densely aggregated thick-walled cells [4,12,26,38,39]. Lastly, “**dematiaceous mycelial fungi**” are those that are stress-tolerant, have dark-colored cell walls, and expansively growing melanized colonies that do not display the morphological characteristics of the previous groups [3,4,26]. Even though the aforementioned groups have their own morphological characteristics, it is often difficult to classify each fungus within only one group, because they have common traits. For instance, some meristematic fungi can also be classified morphologically as black yeasts and vice versa, and some microcolonial fungi can be considered meristematic at the same time [4,26]. This is due to the great growth flexibility of RIF, being able to shift from one growth mode to another according to the needs and stress conditions of the environment which they inhabit [11,26,40]. Several studies have shown that meristematic growth facilitates survival at extreme temperatures and desiccation, and can lead to the formation of microcolonies that reduce energy consumption [3,4,11,26,41]. Instead, black yeasts contribute to the formation of biofilms through their extracellular polymeric substance matrices, which protect them from osmotic stress and make them thermotolerant and highly resistant to antifungal agents. The meristematic growth of RIF is the most common form of chromoblastomycosis in animals and humans (producing muriform propagules in infected tissue), while the production of conidia by budding in the so-called black yeasts is frequently associated with superficial infections, such as *tinea nigra*, and systemic mycoses [24,26,27,37,42].

Moreover, the grouping of RIF under different names according to morphological characteristics is artificial from a taxonomic and evolutionary point of view, as previous phylogenetic studies have shown that they belong to evolutionarily distant orders, namely Capnodiales, Chaetotyriales, Dothideales, and Eurotiales [4,43,44,45,46,47,48]. Recently, a culture-independent molecular technique, i.e., high-throughput amplicon sequencing, has helped to expand our knowledge of the taxonomy of RIF and their role in microbial communities evolving on rocky substrates. However, their application still has some drawbacks, such as contamination with exogenous DNA [49] and limited identification due to the use of short amplicons [50,51,52]. Longer amplicon sequencing, as well as shotgun sequencing, will certainly be two important tools for the study of RIF communities at the species level [52,53].

The main objective of our study was to evaluate, through the use of culture-dependent techniques and a taxonomic polyphasic approach, the biodiversity of the RIF that alter (darken) the facades of buildings in different localities of the Province of Tarragona (Spain), and to determine whether some of them present typical characteristics compatible with extremophilic fungi. In addition, the taxonomic identity of the RIF found can alert us about the potential health risk for the inhabitants of these municipalities if they inhale their spores, as they are taxa that frequently trigger hypersensitivity reactions or cause opportunistic mycoses, especially in immunocompromised individuals.

## 2. Materials and Methods

### 2.1. Sampling Sites

The sampling sites were located in four towns in the Tarragona Province (Catalonia community, Spain): Calafell, Els Pallaresos, Montbrió del Camp, and Reus, with a Csa (Mediterranean hot summer) subtype climate described in the Köppen-Geiger classification [54] and settled on calcareous soils. All locations are surrounded by different sorts of Mediterranean crops (carob trees, hazelnuts, cereals, olive trees, vines, etc.), pine forests, and bushes (https://atlasnacional.ign.es/wane/Suelos (accessed on 12 June 2023)). The town of Reus also has an ornamental flora that comprises a large number of exotic plants, a legacy of nineteenth-century culture. The main meteorological data of the sampled sites are given in Table 1.

### 2.2. Sampling Design

In November 2020, the samples were taken from the rocky exteriors of urban buildings in four towns in the Province of Tarragona. Five samples were taken at five points at each location, except for Els Pallaresos, where ten samples were taken (five in the urban area and five in the industrial one). The sort of samples collected, and their geographical location, are given in Table 2.

The samples were collected with sterile swabs soaked in Ringer’s solution (an isotonic solution that contains NaCl (0.86%), KCl (0.03%), CaCl_2_ (0.01%) and NaHCO_3_ (0.02%) [55], by rubbing an area of 20 cm^2^ (approx.) and then placing the swabs into sterile transport tubes containing 1 mL of Ringer’s solution. In the laboratory, the samples were stored at 4 °C until processed.

### 2.3. Fungal Isolation

Samples were vortexed three times at 1200 RPM for two minutes (leaving five minutes between each operation) and inoculated directly with the swab moistened with the sample, using the technique of loop depletion by duplicate, since the samples were incubated at two different temperatures, 15 °C and 25 °C, in the dark on different culture media. Subsequently, the culture media were placed in 90 mm diameter sterile disposable Petri dishes: potato dextrose agar (PDA; Pronadisa, Madrid, Spain; [56]); potato carrot agar (PCA; 20 g/L potato, 20 g/L carrot, 20 g/L bacteriological agar, 0.2 g/L chloramphenicol; [57]); tap water agar (TWA; 15 g/L bacteriological agar, 0.2 g/L chloramphenicol; [58]); and dichloran-Rose Bengal-chloramphenicol agar (DRBC; Condalab, Madrid, Spain; [59,60]). Fungal colony growth was followed weekly under a stereoscope for up to three months. To obtain the fungal strains, vegetative and reproductive structures were collected using sterile needles (tuberculin/insulin type) and transferred onto PDA and onto oatmeal agar (OA; 30 g/L filtered oatmeal flakes after boiling for one hour, 20 g/L bacteriologic agar, 0.2 g/L chloramphenicol; [61]) in 50 mm diameter sterile disposable Petri dishes, which were incubated at the same temperatures as described earlier.

### 2.4. Phenotypic Characterization

Vegetative and reproductive fungal structures were collected from mature colonies of the fungal strains using sterile hypodermic needles and syringes (tuberculin/insulin type) and deposited onto a drop of 65% lactic acid (as the mounting medium), between slide and cover slide. Then, these structures were observed and measured using an Olympus BH-2 bright field microscope with an eye scale (Olympus Corporation, Tokyo, Japan). For those strains of special interest, the morphology was studied in detail using the slide culture technique according to Egidi et al. [48], which consists of inoculating blocks of 1 cm^2^ of OA and PCA into wet chambers and incubating at 25 °C for up to 12 weeks. Images of the fungal structures were acquired with a DeltaPix Infinity X coupled to the Zeiss Axio Imager M1 microscope (Oberkochen, Germany), using Nomarski (interference contrast) and phase contrast condensers. These images were edited via Adobe Photoshop CS6 v. 13.0 (Adobe Systems, San Jose, CA, USA).

The culture characteristics were documented, growing the strains on malt extract agar (MEA; 30 g/L malt extract, 5 g/L peptone, 15 g/L bacteriologic agar; [61,62]) or MEA 2% (20 g/L malt extract, 15 g/L bacteriologic agar; [63]), OA, PCA, and PDA in 90 mm diameter disposable Petri dishes and incubating at 25 °C for two to four weeks. The colors were described according to Kornerup and Wanscher (1978) [64]. In addition, when necessary, culturing was carried out by inoculating the fungal strain onto sterile plant material (such as oak and palm leaves, filter paper, and pine needles) and into TWA according to Smith et al. [65]. Optimal, minimum, and maximum growth temperatures were determined by growing the strains on PDA at 5, 12, 15, 20, 25, 30, 35, 37, 40, and 45 °C for two weeks.

### 2.5. DNA Extraction, Amplification, and Sequencing

Fungal strains were grown on PDA for one or two weeks at 25 °C. After that, the mycelium was removed by scraping using a sterile scalpel to extract the DNA according to the FastDNA kit protocol (Bio; Vista, CA, USA) plus 50 mg of 425–600 μm size-fractionated glass beads, acid-washed [Sigma] with a FastPrep-24™ instrument (Thermo Savant, Holbrook, NY, USA). The DNA was quantified using a NanoDrop 2000 instrument (Thermo-Scientific, Madrid, Spain). We amplified a fragment of the 28S nrRNA gene (LSU), of the second largest subunit of the RNA polymerase II (*rpb*2), of the β-tubulin (*tub*2), and of the translation elongation factor-1α (*tef*1), and the entire rDNA internal transcribed spacer region (ITS). The primers used are listed in Table 3.

The PCR reactions were performed using EmeraldAmp^®^ GT PCR Master Mix (Takara Bio Inc., Saint-Germain-en-Laye, France) according to the manufacturer’s manual for 25 μL of reaction [75]. In each 25 μL reaction tube, 5 pmol of each primer and 50 ng of template DNA were added. The amplification was carried out using a MyCycler ™Thermal Cycler (Bio-Rad, Feldkirchen, Germany) under the following conditions: initial denaturation temperature of 94 °C for 5 min, 35 cycles of denaturation temperature of 95 °C for 30 s, annealing of the primer at the temperature stipulated in Table 3 for 45 s, primer extension at 72 °C for 120 s, and a final extension step at 72 °C for 7 min. The amplicons were sequenced in both directions with the same primer pair used for amplification at Macrogen Spain (Macrogen Inc., Madrid, Spain). The consensus sequences were obtained using the SeqMan software version 7.0.0 (DNAStar Lasergene, Madison, WI, USA), and then deposited at the European Nucleotide Archive (ENA) (Table S1 ).

### 2.6. Fungal Identification and Phylogenetic Analyses

The nucleotide sequence of each *locus* generated in this study was subjected to a comparison with that at the National Center for Biotechnology Information (NCBI) database using the Basic Local Alignment Search Tool [76] (BLAST; https://blast.ncbi.nlm.nih.gov/Blast.cgi (accessed on 12 December 2023)). Fungal strains were identified at the species level when the ITS sequences displayed a level of identity ≥ 98% with those of ex-type and/or reference strains in the database.

Phylogenetic analyses were performed using the phylogenetic markers LSU (for general analysis), *rpb2* (specifically for the genus *Neocatenulostroma*), and ITS (for the rest of the genera). The nucleotide sequences of the markers of the most phylogenetically related taxa were retrieved from GenBank (https://www.ncbi.nlm.nih.gov (accessed on 14 May 2023); see Table S2). The nucleotide sequences were aligned separately using the ClustalW algorithm [77] in the MEGA software v. 7.0 [78] and manually adjusted using the same software. Phylogenetic reconstructions were made for each phylogenetic marker by maximum likelihood (ML) and Bayesian inference (BI) with RAxML [79] in CIPRES web (https://www.phylo.org/ (accessed on 14 May 2023)) [80] and MrBayes 3.2.6 [81], respectively. The best substitution model for each gene matrix was estimated using MrModelTest v. 2.3.25 [82]. For ML analyses, the nearest-neighbour interchange was used as the heuristic method for tree inference. Support for internal branches was assessed with 1000 ML bootstrapped pseudo-replicates. A bootstrap support (BS) of ≥70 was considered significant. For BI analyses, Markov chain Monte Carlo (MCMC) [83] sampling was carried out with four million generations, with samples taken every 1000 generations. The 50% majority rule consensus trees and posterior probability values (PP) were calculated after removing the first 25% of the resulting trees for burn-in. A PP value of ≥0.95 was considered significant.

### 2.7. Physiological Characterization of the Strains of Interest

Eleven strains, mostly producers of yeast-like cells, were grown in MEA or PDA and incubated at 25 °C for 47 days, depending on their growth rates. Then, inocula were prepared by aseptically adding small colony portions by a loop in sterile water into 16 mm diameter glass tubes with a screw cap until a final concentration of 5 × 10^5^ CFU/mL was reached, or an Optical Density (OD) of OD_520_ 0.20–0.25, filtering through sterile gauze to remove hyphae if necessary. The evaluation of the nutritional abilities and tolerance tests were based on the methods described by van der Walt and Yarrow [84] and adapted for black yeasts by de Hoog et al. [85] and Wollenzien et al. [86]. The pH tolerance was checked according to Uribe [87]. The assimilation of carbon sources was carried out according to Schwarz et al. [88] and Alvarez et al. [89], using the commercial kit API 50 CH (bioMérieux, Marcy, l’Etoile, France), the inoculum was prepared in a Yeast Nitrogen Base (YNB; 6.7 g/L [BD Difco, Madrid, Spain], 0.5 g/L L-chloramphenicol, 0.05% *w/v* bacteriologic agar), and the results were read every week. All assimilation assays (×triplicate) were incubated for up to 2 or 3 weeks at 25 °C and were either stationary or horizontally shaken, as indicated by the procedure.

## 3. Results

### 3.1. Phenotypic and Molecular Identification of the Fungal Strains

Using a polyphasic approach, we identified a total of 224 fungal strains (32 from Calafell, 63 [urban area] and 58 [industrial area] from Els Pallaresos, 14 from Montbrió del Camp, and 57 from Reus) belonging to 41 genera and 64 species. However, not all the strains were molecularly identified (Table S1).

The strains belonged to the orders previously reported as RIF with Pleosporales being the most abundant and recovered from all sampled sites. In addition, we recovered representatives of the orders Botryosphaeriales, Capnodiales, Cladosporiales, Chaetotyriales, Coniochaetales, Dothideales, Hypocreales, Lichenostigmatales, Mucorales, Sordariales, and Xylariales [1].

**Figure 1:**
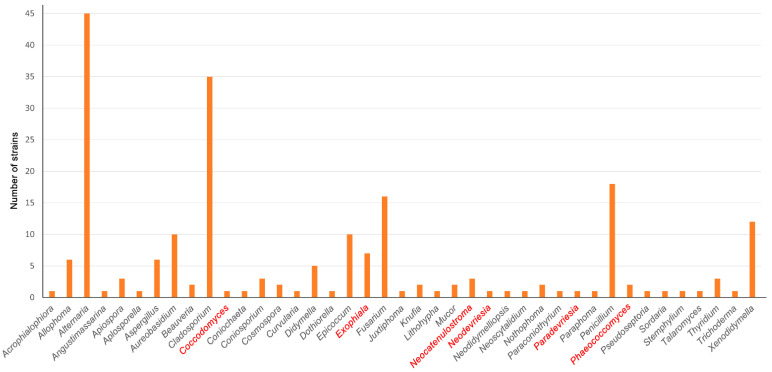
Relative abundance of the genera found in the samples analyzed (red color indicates the genera where new species are reported).

Figure 1 shows the relative abundance (based on the number of strains) of the genera recovered from the samples. *Alternaria* (45) was the most abundant, followed by *Cladosporium* 35), *Penicillium* (18), *Fusarium* (15), *Xenodidymella* (12), *Aureobasidium* (10), and *Epicoccum* (10). Several genera had only one strain, i.e., *Angustimassarina*, *Apiospora*, *Beauveria*, *Coniochaeta*, *Cosmospora*, *Dothiorella*, *Juxtiphoma*, *Neodidymelliopsis*, *Paraconiothyrium*, *Paraphoma,* and *Pseudoseptoria*. Additionally, we isolated several previously unknown species belonging to the genera *Exophiala*, *Neocatenulostroma*, *Neodevriesia, Paradevriesia,* and *Phaeococcomyces* ([90]) (Figure 1, written in red), as well as the new genus *Coccodomyces*; all of them were isolated from Els Pallaresos and are suggested here as new taxa.

**Figure 2:**
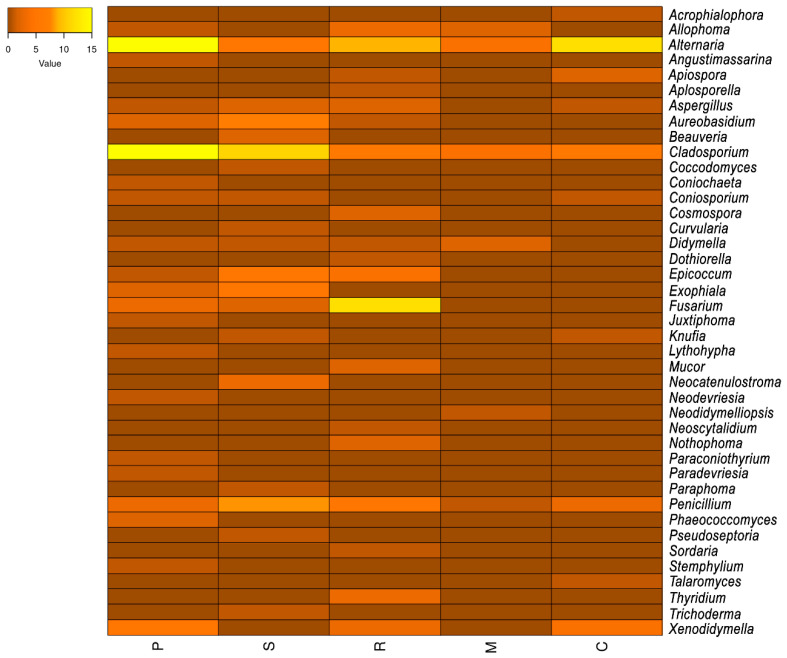
Heatmap showing the relative abundance and distribution of fungal genera among studied sites. (P, Els Pallaresos, urban area; S, Els Pallaresos, industrial area; R, Reus; M, Montbrió del Camp; C, Calafell).

The relative abundance of fungal genera was also compared among the sampled sites, and the results are shown as a heatmap (Figure 2). Thus, Montbrió del Camp and Calafell had the least fungal diversity, while Els Pallaresos had the highest biodiversity, followed by Reus. The most abundant genera, *Alternaria*, *Cladosporium*, and *Penicillium*, were isolated from all locations, though many of the genera were isolated from only one location (Figure 2). Regarding the preference for growing on certain substrates, it was found that *Acrophialophora*, *Angustimassarina*, *Aplosporella*, *Beauveria*, *Cosmospora*, *Curvularia*, *Dothiorella*, *Juxtiphoma*, *Lythohypha*, *Mucor*, *Neodidymelliopsis*, *Neoscytalidium*, *Nothophoma*, *Paradevriesia*, *Paraphoma*, *Pseudoseptoria*, *Sordaria*, *Stemphylium, Talaromyces*, and *Thyridium* only grew on concrete, while *Coccodomyces*, *Neodevriesia*, *Paraconiothyrium,* and *Phaeococcomyces* were found on metal structures, and *Necatenulostroma* and *Trichoderma* on a PVC pipe.

### 3.2. Phylogeny

The accession numbers of the nucleotide sequences obtained in this study are listed in Table S1, while those retrieved from NCBI databases to build the phylogenetic trees are listed in Table S2.

**Figure 3:**
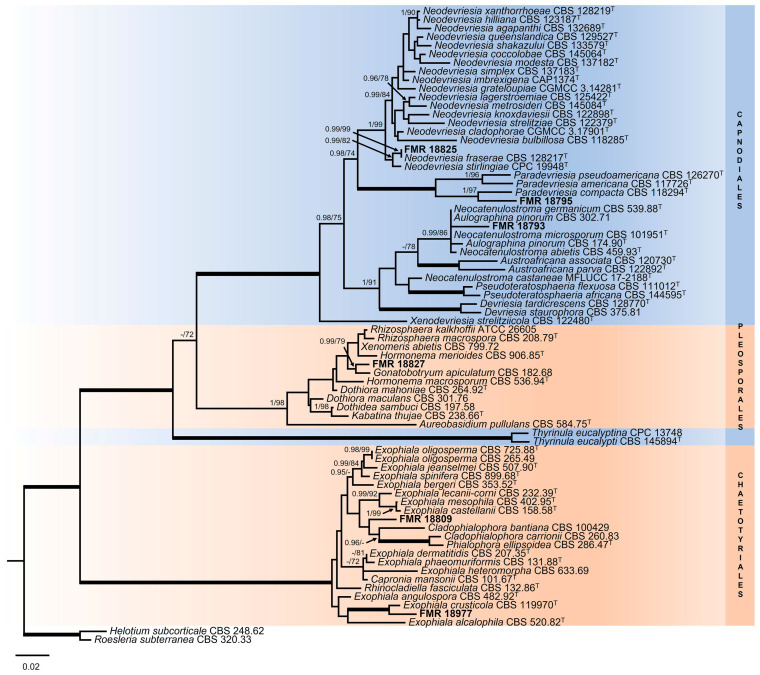
Maximum likelihood tree based on LSU alignment (899 pb) of the sequences from our strains and retrieved from the GenBank. Bayesian posterior probabilities (PP) equal to or above 0.95 and the RAxML bootstrap support values (BS) ≥ 70% are presented at the nodes (PP/BS). Thickened branches indicate full support (PP = 1 and BS = 100%). *Roesleria subterranea* (CBS 320.33) and *Helotium subcorticale* (CBS 248.62) were used as outgroups. Strains corresponding to the potential new species are indicated in **bold**. ^T^ represents the ex-type strain of the species.

It is important to mention that the *rpb*2 *locus* was not always correctly amplified. The general LSU phylogenetic tree (Figure 3), based on 899 positions including gaps, confirmed that our strains FMR 18793, FMR 18795, and FMR 18825 belonged to the order Capnodiales, while FMR 18977 and FMR 18809 were located within the order Chaetotyriales, and FMR 18827 in the order Pleosporales, all of these being considered as potential new taxa.

#### 3.2.1. Order Dothideales

**Figure 4:**
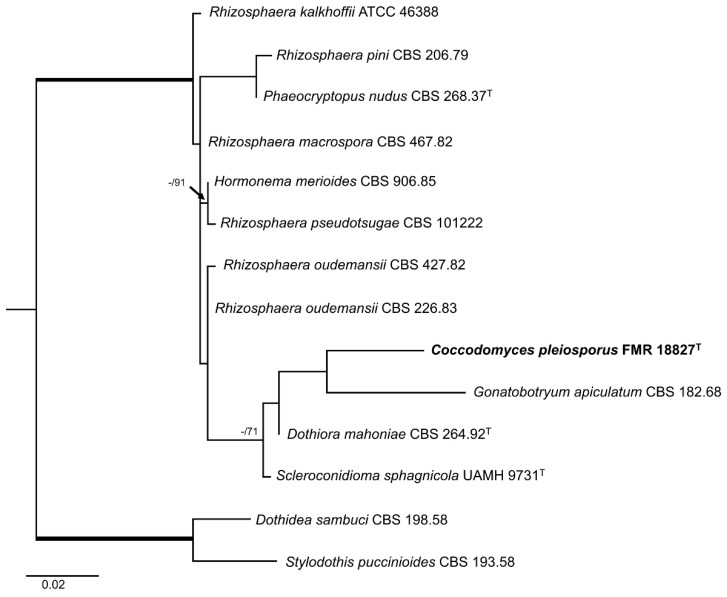
Maximum likelihood tree based on ITS alignment (575 bp) of the sequences from our strain FMR 18827 and those retrieved from the GenBank. Bayesian posterior probabilities (PP) equal to or above 0.95 and the RAxML bootstrap support values (BS) ≥ 70% are presented at the nodes (PP/BS). Thickened branches indicate full support (PP = 1 and BS = 100%). *Dothidea sambuci* (CBS 198.58) and *Stylodothis pucciniodes* (CBS 193.58) were used as outgroups. The new genus is indicated in **bold**. ^T^ represents the ex-type strain of the species.

For the strain FMR 18827, the ITS alignment included 12 ingroups and two outgroups (*Dothidea sambuci* CBS198.58 and *Stylodothis pucciniodes* CBS 193.58) with a total of 575 characters (including gaps). The BI and ML showed similar topology and congruent results. In the phylogenetic tree (Figure 4) FMR 18827 was located within the order Dothideales, in the same linage (fully supported) as *Rhizosphaera* spp., *Hormonema merioides* CBS 906.85, and *Phaeocryptopus nudus* CBS 268.37, and in the same terminal clade (0.70 PP/71% BS) as *Gonatobotryum apiculatum* CBS 182.68, *Dothiora mahoniae* CBS 264.92, and *Scleroconidioma sphagnicola* UAMH 9731.

#### 3.2.2. Genus *Exophiala*

**Figure 5:**
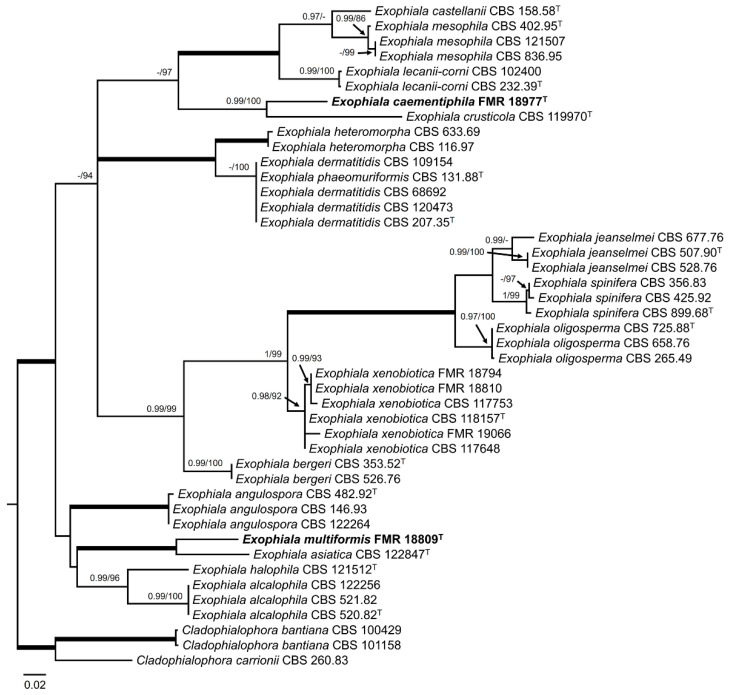
Maximum likelihood tree based on ITS alignment (586 bp) of the sequences of our strains (FMR) and those retrieved from the GenBank (CBS). Bayesian posterior probabilities (PP) equal to or above 0.95 and the RAxML bootstrap support values (BS) ≥ 70% are presented at the nodes (PP/BS). Thickened branches indicate full support (PP = 1 and BS = 100%). *Cladophialophora bantiana* (CBS 101158 and CBS 100429) and *Cl. carrioni* (CBS 260.83) were used as outgroups. The new species are indicated in **bold**. ^T^ represents the ex-type strain of the species.

For *Exophiala* spp., the ITS alignment comprised 41 ingroups and three outgroups (*Cladophialophora bantiana* CBS 101158 and CBS100429, and *Cladophialophora carrioni* CBS 260.83) with 586 characters including gaps. The BI and ML showed similar topology and congruent results. In the phylogenetic tree (Figure 5) the strains FMR 18794, FMR 18810, and FMR 19066 were placed in a well-supported (0.98 PP/92% BS) terminal clade corresponding to *E. xenobiotica*. On the other hand, the strain FMR 18977 was placed in a well-supported (0.99 PP/100% BS) terminal clade together with the type strain of *E. crusticola* (CBS 119970), but as a different species (BLAST Id = 88%), while the strain FMR 18809 was located in other well-supported (1 PP/100% BS) terminal clade together the type strain of *E. asiatica* (CBS 122847), but as a different species (BLAST Id = 88%).

#### 3.2.3. Order Capnodiales

**Figure 6:**
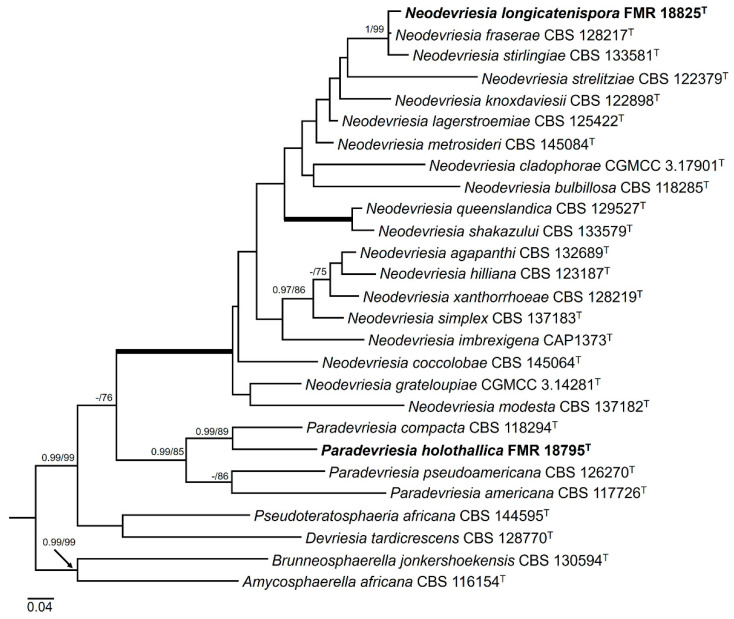
Maximum likelihood tree based on ITS alignment (514 pb) of the sequences of our strains (FMR) and those retrieved from the GenBank. Bayesian posterior probabilities (PP) equal to or above 0.95 and the RAxML bootstrap support values (BS) ≥ 70% are presented at the nodes (PP/BS). Thickened branches indicate full support (PP = 1 and BS = 100%). *Amycosphaerella africana* (CBS 116154) and *Brunneosphaerella jonkershoekensis* (CBS 130594) were used as outgroups. The new species proposed in this study are indicated in **bold**. ^T^ represents the ex-type strain of the species.

For the genera *Neodevriesia* and *Paradevriesia*, the ITS sequence alignment included 25 ingroups and two outgroups (*Amycosphaerella africana* CBS 116154 and *Brunneosphaerella jonkershoekensis* CBS 130594) with 514 characters including gaps. The BI analysis showed a similar tree topology and congruent results compared to those obtained in ML. The phylogenetic inference (Figure 6) shows that our strain FMR 18825 forms a well-supported (1 PP/99% BS) terminal clade, together with *N. fraserae* (CBS 128217) (BLAST Id = 99%) and *N. stirlingiae* (CBS 133581) (BLAST Id = 98%), within a main fully supported clade corresponding to all species of *Neodevriesia*. Moreover, the BLAST search for the strain FMR 18825 against *N. fraserae* and *N. stirlingiae* indicated a low similarity for *tub2* (92% and 90%, respectively) and for *rpb2* (94% and 93%, respectively). The main clade corresponding to the species of *Paradevriesia* (0.99 PP/85% BS) included our strain FMR 18795 (BLAST Id = 92%), which was placed as a different species in a branch (0.99 PP/89% BS) with the type strain of *P. compacta*.

**Figure 7:**
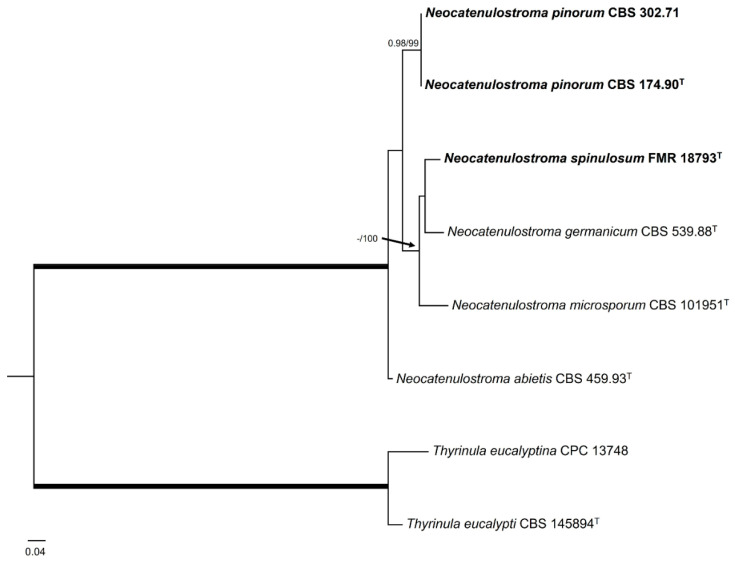
Maximum likelihood tree based on *rpb*2 alignment (910 pb) of the sequences of our strain FMR 18793 and those retrieved from the GenBank. Bayesian posterior probabilities (PP) equal to or above 0.95 and the RAxML bootstrap support values (BS) ≥ 70% are presented at the nodes (PP/BS). Thickened branches indicate full support (PP = 1 and BS = 100%). *Thyrinula eucalyptina* (CPC 13748) and *Thyrinula eucalypti* (CBS 145894) were used as outgroups. The new species/combinations are indicated in **bold**. ^T^ represents the ex-type strain of the species.

Regarding the genus *Neocatenulostroma,* the LSU phylogenetic tree (Figure 3) revealed that all species were placed in a well-supported clade (0.99 PP/86% BS), which also included *Aulographina pinorum* (CBS 174.90 and CBS 302.71) and our strain FMR 18793, with the genus *Austroafricana* (*A. parva* and *A. associata*) as a sister clade, and placed *N. castenae* (MFLUCC 17-2188) [91] outside these clades. In order to solve the genera and species boundaries, a *rpb2* phylogenetic tree was built. The *rpb2* alignment comprised eight ingroups with a total of 910 characters, including gaps, and with *Thyrinula eucalyptina* (CPC 13748) and *Thyrinula eucalypti* (CBS 145894) as outgroups. The BI and ML showed similar topology and congruent results. The *rpb2* phylogenetic tree (Figure 7) confirmed what was observed in the LSU phylogeny: On the one hand, FMR 18793 was placed as a new species of the genus, with *N. germanicum* as its phylogenetically closest species and together forming a sister clade with *N. microsporum*. On the other hand, *N. pinorum* was included in the genus *Neocatenulostroma*. Because *N. castaneae* lacks the *rpb2* nucleotide sequence, it could not, unfortunately, be included in such phylogenetic analysis.

### 3.3. Taxonomy

*Coccodomyces* Sastoque, Cano and Stchigel, gen. nov. Mycobank MB841929.

*Etymology*: From Latin *coccum*-, grain, granule, berry, seed, and Latin < Greek -*μύκης*, mushroom, fungus.

*Classification*—*Incertae sedis*, Dothideales, Pezizomycotina, Ascomycota.

*Colonies* spreading, flat, crateriform or umbonated, margins filiform due to the submerged mycelium, becoming black and leathery with age. *Mycelium* is composed of septate, hyaline, and thin-walled hyphae when young, becoming dark brown, thick-walled, and torulose with age, frequently ending in long terminal non-septate segments. *Hyphae* hyaline to copper-brown, composed of cylindrical, sub-cylindrical to globose cells, thick-walled when pigmented, increasing the degree of constriction and the number of transversal septa with age, forming also additional longitudinal and oblique septa; additionally, there are globose cells produced by the blowing-out of the pre-existent cells, giving a granulose look to the hyphae. *Conidiophores* absent. *Conidiogenous cells* integrated to the hyphae, mono- to polyblastic, bearing lateral denticles (conidiogenous *loci*). *Conidia* holoblastic, one-celled, produced asynchronously in slimy masses, smooth- and thin- to thick-walled, ellipsoidal to globose, hyaline at first, becoming dark brown with age, developing in new conidiogenous cells producing holoblastic conidia by budding, but also forming two-celled conidia by the development of a medial septum which is always constricted due to the swelling of both cells, which also forms in new conidiogenous cells producing inflated, one- or two-celled dark brown conidia in short chains, which can also develop additional longitudinal and oblique septa. *Sexual morph* unknown.

**Figure 8:**
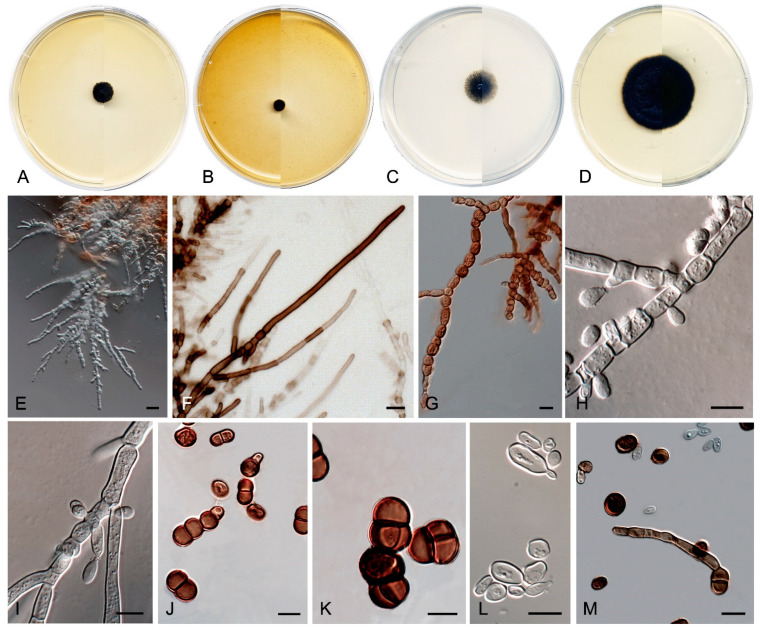
*Coccodomyces pleiosporus* FMR 18827. (**A**–**D**) Colony on MEA, OA, PCA, PDA (after 2 wks at 25 ± 1 °C; surface, left; reverse, right). (**E**–**G**) Isodiametric and torulose hyphae, hyaline to copper-brown colored, with terminal aseptate segments. (**H**,**I**) Conidiogenous cells and holoblastic conidia. (**J**–**L**) Formation of new conidia by septation of pre-existent conidia (**J**,**K**) or by budding cells (**L**,**M**) Conidial germination. DIC Nomarski. Scale bars = 10 μm.

*Coccodomyces pleiosporus* Sastoque, Cano and Stchigel, sp. nov. MycoBank MB841950. Figure 8.

*Etymology*. From Greek *πλειο*-, more, -*σπόρος*, spore, due to the production of conidia which are variable in shape and number of cells.

*Description*—On potato dextrose agar after two weeks at 25 °C—*Mycelium* composed of septate, hyaline, and thin-walled branching hyphae when young, becoming dark brown, thick-walled, and torulose with age, 4–8 µm wide (Figure 8E–G), ending in terminal aseptate segments of up to 300 µm long (Figure 8F). *Hyphae* hyaline to copper-brown, guttulate when young, thick-walled when pigmented, composed of cylindrical, subcylindrical to globose cells, 5–20 × 3–7 µm, increasing in the degree of constriction at and the number of transversal septa with age, and then forming additional longitudinal and oblique septa; additional globose cells produced by the blowing-out of the pre-existent cell, giving a granulose look to the hyphae (Figure 8G). *Conidiophores* absent. *Conidiogenous cells* integrated to hyphae, smooth or nearly so, thin-walled, prismatic, barrel-shaped, or globose, 5–20 × 3–10 µm, occasionally arising as globose lateral cells, mono- to polyblastic, bearing lateral denticles of 1–3 × 0.5–2 µm (Figure 8H,I). *Conidia* holoblastic, one-celled, hyaline at first, becoming dark brown with age, produced asynchronously in slimy masses, smooth- and thin- to thick-walled, ellipsoidal to globose, 6–13 × 2–7 µm, covered with a pigmented mucous layer with age and attaching the conidial mass to the hyphae; conidia also developing in new conidiogenous cells producing holoblastic conidia by budding but also forming two-celled conidia by the development of a medial septum which is always constricted due to the swelling of both cells, which also develop in new conidiogenous cells producing inflated, one- or two-celled dark brown conidia, 11–22 × 6–10 µm, disposed in short chains, which can also develop additional longitudinal and oblique septa (Figure 8H–L). *Chlamydospores*, *endoconidia*, and *sexual morph* not seen.

*Culture characteristics*—(After 14 days at 25 °C, Figure 8A–D) Colonies reaching 11 mm diameter on MEA, 6 mm on OA, 24 mm on PCA, and 37 mm on PDA, spreading, flat, glistening, dry, and slightly umbonated or crateriform, with filiform margins due to the presence of submerged hyphae mostly on PCA, without aerial mycelium, becoming coriaceous with age, surface black (6F3) (according to Kornerup and Wanscher [64]) on all culture media tested, with pale yellow (2A3) margins (surface and reverse) on MEA and PDA; reverse, grey (29F1) and black (6F3) on MEA, black (6F3) on OA and PCA, and nickel-green (27F3) and black (6F3) on PDA. Minimum, optimum, and maximum temperature of growth: 5 °C, 25 °C, and 30 °C, respectively.

*Type*—SPAIN, Tarragona Province, Els Pallaresos, isolated from a blackened metal fence of an industrial warehouse N 41°10′34.2″ E 1°16′14.4″, 20 November 2020, J. F. Cano-Lira and A. M. Stchigel, isol. A. P. Sastoque (holotype CBS H-24941, ex-type FMR 18827 = CBS 149014; ITS and LSU sequences GenBank OW273979 and OW370575, respectively).

*Diagnosis*—Morphologically, *Coccodomyces pleiosporus* differs from *Gonatobotryum apiculatum*, the closest species, in its diameter and the shape of the colony on PDA, being filiform and coriaceous. *C. pleiosporus* has torulose and hyaline to copper-brown hyphae with terminal aseptate segments up to 300 µm long, an absence of conidiophores, conidiogenous cells integrated into the hyphae, conidia holoblastic, but also producing holoblastic conidia by budding or forming two-celled conidia by the development of a medial septum that is always constricted due to the swelling of both cells. By contrast, *G. apiculatum* presents conidiogenous ampullae with echinulate cicatrized scars, its conidiophores are erect or flexuous, unbranched, and nodose, and its conidia are catenate and aseptate [92]. The sexual morph of *C. pleiosporus* was not observed.

*Notes*—Based on a mega BLAST search of NCBIs GenBank nucleotide database, the closest hit using the **ITS** sequence was *Scleroconidioma sphagnicola* (strain UAMH 9731, GenBank NR_121294; identities = 490/507 (96.65%), gaps 2/507 (0%)), and the closest hit using the **LSU** sequence was *Plowrightia abietis* (strain ATCC 24339, GenBank EF114703; Identities = 819/828 (98.91%), gaps 0/828 (0%)).

**Figure 9:**
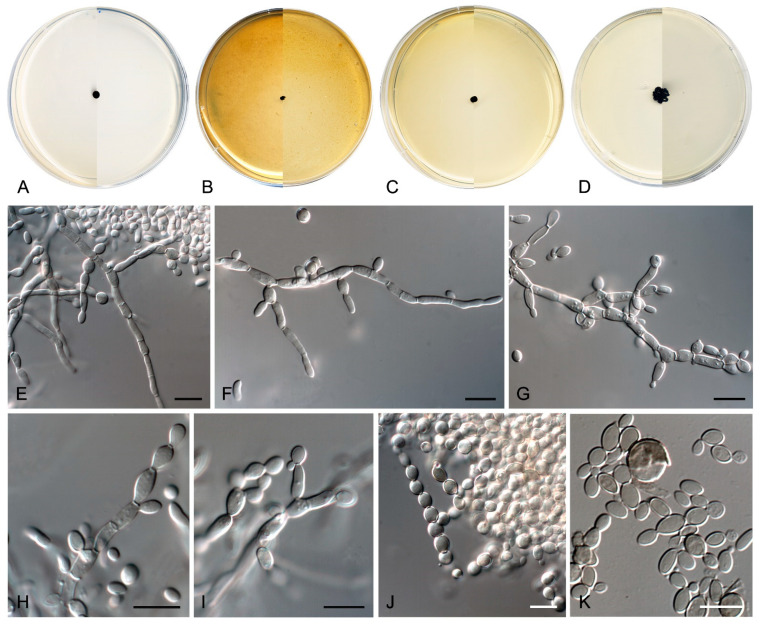
*Exophiala caementiphila* FMR 18977. (**A**–**D**) Colony on MEA, OA, PCA, PDA (after 2 wks at 25 ± 1 °C; surface, left; reverse, right). (**E**–**G**) Mycelium and anelidic conidiogenous cells (**H**,**I**) Enteroblastic conidia. (**J**) Conidia chains. (**K**) Yeast-like cells. DIC Nomarski. Scale bars = 10 μm.

*Exophiala caementiphila* Sastoque, Stchigel, and Cano, sp. nov. Mycobank MB 849258. Figure 9.

*Etymology*. From Latin *caementum-*, cement, and from Latin < Greek -*φιλíα*, friendship, because the substrate on the fungus develops.

*Classification*—Herpotrichiellaceae, Chaetothyriales, Chaetothyriomycetidae, Eurotiomycetes, Pezizomycotina, Ascomycota.

*Description*—On potato dextrose agar after two weeks at 25 °C—*Mycelium* scarce, submerged, composed of pale olivaceous-brown to olivaceous-brown, septate, branching, smooth- and thin-walled hyphae, consisting of cylindrical or torulose cells, 2–4 μm wide (Figure 9E). *Conidiogenous cells* annellidic, mono- to polyblastic, integrated to the hyphae or discrete, then laterally disposed, pale olivaceous-brown to olivaceous-brown, cylindrical, sub-cylindrical, ellipsoidal to broadly ovoid, 4–12 × 2–4 μm (Figure 9F–H). *Conidia* enteroblastic, non-septate, subhyaline to pale olivaceous-brown, smooth- and thin-walled, cylindrical with rounded ends, ellipsoidal, ovoid to nearly globose, 2–8 × 2–4 μm, often forming chains. *Yeast-like cells* very abundant, non-septate, subhyaline to olivaceous brown, smooth- and thin-walled, ellipsoidal to ovoid or nearly globose, 2–7 × 2–5 μm, developing secondary conidia to form long chains (Figure 9I–K). *Chlamydospores* and *sexual morph* not seen.

*Culture characteristics*—(After 14 days at 25 °C, Figure 9A–D) Colonies reaching 3.0 mm diameter on MEA and PCA, 1.5 mm diameter on OA, and 5.0 mm diameter on PDA, circular, margins entire and regular, convex to slightly pulvinate, mucoid at first, soon becoming smooth to verrucose, dry and glistening, cerebriform with age on MEA, OA, and PDA, but mucoid on PCA. Surface and reverse black (6F3) on MEA, OA, and PDA, and black (6F3) with dark brown (6F4) margins on PCA. Minimum, optimum, and maximum temperature of growth: 5 °C, 15−25 °C, and 25 °C, respectively.

*Type*—Spain, Tarragona Province, Els Pallaresos, isolated from a blackened wall of an industrial warehouse N 41°10′34.8″ E 1°16′13.4″, 20 November 2020, coll. J. F. Cano-Lira and A. M. Stchigel, isol. A. P. Sastoque (holotype CBS H-25346, ex-type FMR 18977 = CBS 150902; ITS, LSU, *tef* and *tub2* sequences GenBank OX380503, OX380504, OX380501, and OX380502, respectively).

*Diagnosis*—*Exophiala caementiphila* exhibits the most important features of the genus, such as annelidic conidiogenous cells, the presence of budding cells and torulose hyphae, as well as the formation of chains of conidia (its cladophialophora-like synanamorph). However, *E. caementiphila* differs from *E. crusticola*, the phylogenetically closest species, in having scarce mycelium, integrated or discrete conidiogenous cells, and conidia forming chains [93]. Physiologically, *E. crusticola*, in contrast to *E. caementiphila,* is able to assimilate arginine and ornithine, but not galactose, inulin, maltose, raffinose, and sucrose.

*Notes*—Based on a mega BLAST search of NCBIs GenBank nucleotide database, the closest hit using the **ITS** sequence was *E. crusticola* (strain CBS 119970, GenBank NR_165997; Identities = 493/569 (86.24%), gaps = 41/569 (7%)), and using the **LSU** sequence it was the same strain (GenBank MH874623; Identities = 424/434 (97.70%), gaps = 0/434 (0%)).

**Figure 10:**
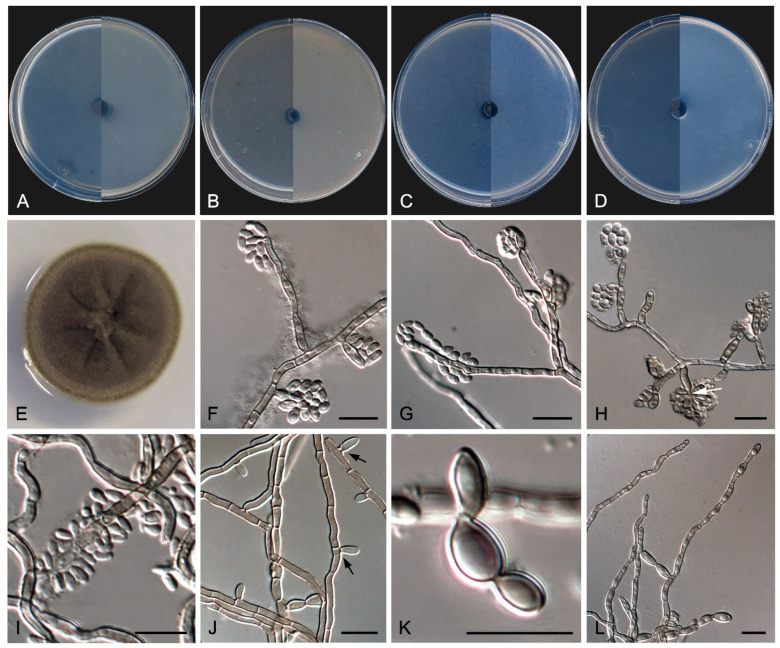
*Exophiala multiformis* FMR 18809. (**A**–**D**) Colony on MEA, OA, PCA, PDA (after 2 wks at 25 ± 1 °C; surface, left; reverse, right). (**E**) Colony appearance on PDA at 25 ± 1 °C. (**F**–**H**) Macro, semi-micro, and micronemotous conidiphores and annelidic conidiogenous cells (white arrow). (**I**) Semi-micronematous conidiophore with sympodial proliferation. (**J**) Conidiogenous cells integrated into the hyphae (black arrows). (**K**) Yeast-like cells. (**L**) Cylindrical and torulose hyphae. DIC Nomarski. Scale bars = 10 μm.

*Exophiala multiformis* Sastoque, Cano, and Stchigel, sp. nov. MycoBank MB842313. Figure 10.

*Etymology*. From Latin *multi*-, many, and -*formis*, shapes, because of the diversity of reproductive structures produced.

*Classification*—Herpotrichiellaceae, Chaetothyriales, Chaetothyriomycetidae, Eurotiomycetes, Pezizomycotina, Ascomycota

*Description*—On potato dextrose agar after two weeks at 25 °C—*Mycelium* abundant, composed of olivaceous-brown, smooth- and thin-walled septate hyphae, 1–3 μm wide, surrounded by a partially soluble dark pigment (Figure 10L). *Conidiophores* of three kinds: micronematous, reduced to an olivaceous brown to brown annellidic conidiogenous cell integrated to the vegetative hyphae or discrete, if discrete sub-cylindrical, cylindrical to flask-shaped, 4–8 × 2–4 μm; semi-micronematous, arising as short lateral branches from the vegetative hyphae, one-celled, mostly septate at the base, cylindrical but constrained at the septum, 5–10 × 2–4 μm, truncated at both ends, bearing one, or rarely two, annellidic conidiogenous cells which can also proliferate percurrently to form long chains of additional conidiogenous cells; macronematous, erect, straight, unbranched, smooth- and thin-walled, brown but becoming paler towards the apex, continuous to 2-septate, up to 40 μm long, bearing a terminal integrated conidiogenous cell proliferating sympodially, resulting in one or two conspicuous conidiogenous *loci*, cylindrical or nearly so, 6–15 × 3–4 μm, scars not seen (Figure 10F–J). *Conidia* non-septate, subhyaline to pale olivaceous-brown, smooth- and thin-walled, ellipsoidal to ovoid, 2–6 × 1–4 μm. *Budding cells* barely present, morphologically like those conidia from annellidic conidiogenous cells, 3–6 x 2.5–4.5 μm (Figure 10K). *Chlamydospores* and *sexual morph* not seen.

*Culture characteristics*—(After 14 d at 25 °C, Figure 10A–E) Colonies reaching 8.5 mm diameter on MEA, 7 mm on OA, 8 mm on PCA, and 9 mm on PDA, restricted, circular, margins entire, flat to slightly convex or elevated at the center, velvety on MEA and PDA, but brightening (due to the production of yeast-like cells) at the center on OA and PCA. Surface color on all culture media tested dull green (29E4) with light green margins (30E6), and reverse bronze green (30F3). Minimum, optimum, and maximum temperature of growth: 15 °C, 25 °C, and 30 °C, respectively.

*Type*—Spain, Tarragona Province, Els Pallaresos, isolated from a blackened metal fence of an industrial warehouse N 41°10′34.2″ E 1°16′14.4″, 20 November 2020, coll. J. F. Cano-Lira and A. M. Stchigel, isol. A. P. Sastoque (holotype CBS H24940, ex-type FMR 18809 = CBS 149013; ITS and LSU sequences GenBank OU624180 and OU624179, respectively.

*Diagnosis*—*Exophiala multiformis* differs from *E. asiatica*, the phylogenetically closest species, by its larger and thinner annellidic conidiogenous cells (4–8 × 2–4 μm in *E. multiformis* vs. 4.5–6.0 × 4–5 μm in *E. asiatica*), the presence of torulose hyphae (nearly absent in *E. asiatica)* and the bigger annelloconidia (2–6 × 1–4 μm in *E. multiformis* vs. 3–4.5 × 1–2 μm in *E. asiatica*), and by the presence of well-developed conidiophores whose apices proliferate sympodially (absents in *E. asiatica*). Moreover, *E. asiatica* can grow at up to 40 °C, while *E. multiformis* only grows at up to 30 °C [94].

*Notes*—Based on a Mega BLAST search of NCBIs GenBank nucleotide database, the closest hit using the **ITS** sequence was *E. asiatica* (strain CBS 122847, GenBank MH863242; Identities = 458/522 (87.74%), 19/522 (3%)), and using the **LSU** sequence it was *Fonsecaea brasiliensis* (strain CBS 127815, GenBank MH877954; Identities = 748/766 (97.70%), 1/766 (0%)).

*Neocatenulostroma* Quaedvlieg and Crous.

**Figure 11:**
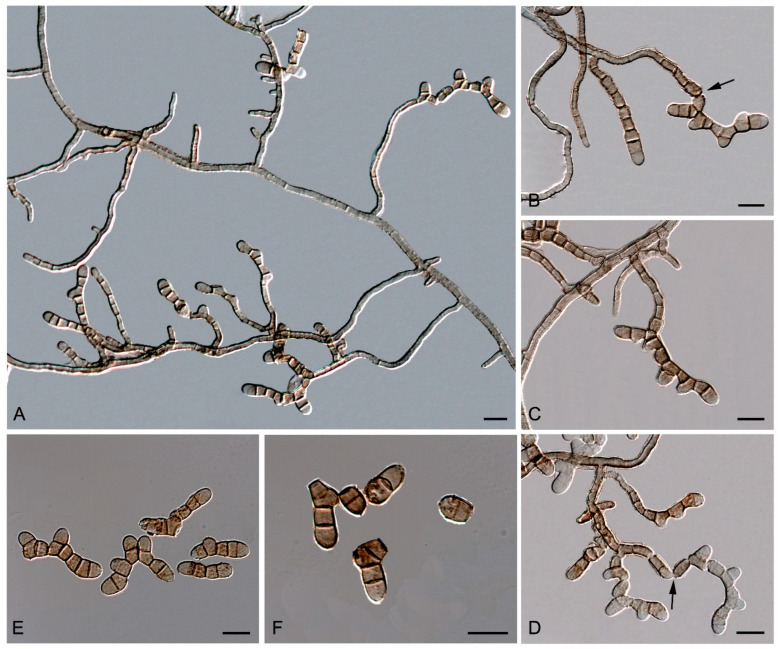
*Neocatenulostroma abietis* CBS 459.93. (**A**) Overview of conidiophores arising laterally from the mycelium. (**B**–**D**) Macronematous conidiophores with catenated thallic–arthric conidia disarticulated by schizolytic secession (arrows). (**E**,**F**) Thallic–arthric multi-septate conidia, with transverse and occasionally oblique septa, catenate, variously shaped, straight, or curved. DIC Nomarski. Scale bars = 10 μm.

*Type species*—*Neocatenulostroma abietis* (Butin and Pehl) Quaedvl. and Crous. Figure 11.

*Classification*—Teratosphaeriaceae, Capnodiales, Dothideomycetes, Pezizomycotina, Ascomycota.

*Emended description*—*Mycelium* composed of pale brown to brown, septate, branched, smooth- and thin-walled hyphae (Figure 11A). *Asexual state* consisting of macronematous, mainly straight, caespitose, short, smooth-walled, olivaceous-brown, closely packed conidiophores, emerging laterally from the mycelium through a stoma or forming sporodochia; *conidia* thallic–arthric (Figure 11B,D), olivaceous to red-brown, multi-septate, with transverse and occasionally oblique septa (Figure 11E,F), catenate, in branched chains, with secondary meristematic development, variously shaped (ellipsoidal, cylindrical, Y-shaped, or irregularly-shaped), straight or curved, with truncated to rounded ends, secession schizolytic (Figure 11B–F). *Sexual state* consisting of amphigenous, immersed, substomatal, subepidermal ascomata, with a small papilla or not papillated, globose to subglobose, with a periphysate central ostiole, peridium comprising in two layers, outer layer thick, brown, with *textura angularis*, inner layer thin and hyaline; *asci* 8-spored, bitunicate, obclavate to globose; *ascospores*, medially 1-septate, hyaline to pale brown, broadly fusiform with obtuse apices, eguttulate.

**Figure 12:**
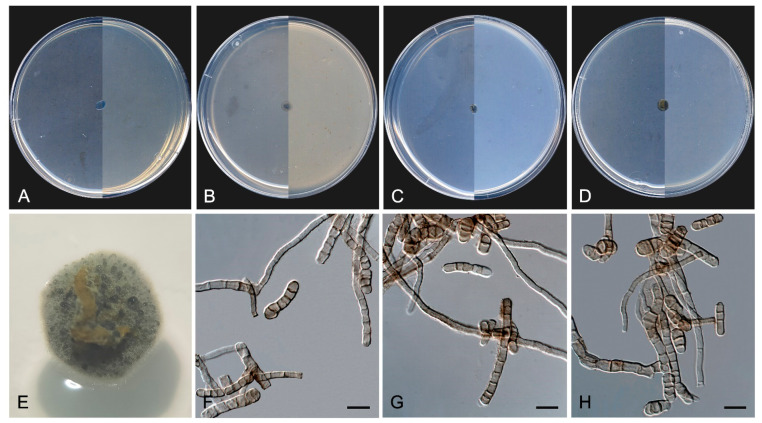
*Neocatenulostroma pinorum* CBS 174.90. (**A**–**D**) Colony on MEA, OA, PCA, PDA (after 2 wks at 25 ± 1 °C; surface, left; reverse, right). (**E**) Colony appearance on PDA. (**F**–**H**) Fertile hyphae from which arthroconidia are released by schizolytic secession. Conidia smooth, 0–4 septa, straight to slightly curved. DIC Nomarski. Scale bars = 10 μm.

*Neocatenulostroma pinorum* (Arx and E. Müll.) Sastoque, Stchigel, and Cano, comb. nov. Mycobank MB326815. Figure 12.

*Basionym*—*Aulographina pinorum* (Desm.) Arx and E. Müll., Sydowia 14: 332 (1960)

≡ *Aulographum pinorum* Desm., Annls Sci. Nat., Bot., sér 2 10: 314 (1838)

*Description*—On oatmeal agar after two weeks at 25 °C—*Mycelium* consisting of septate, branching, olivaceous brown to brown, smooth- and thin-walled, 2–4 µm wide hyphae (Figure 12F–H). *Asexual state* (produced in vitro) consisting of fertile hyphae arising laterally from the mycelium, forming intercalary arthroconidia of thickened walls; *arthroconidia* 0–4-septate, olivaceous brown to brown, smooth- and thick-walled, sub-cylindrical to cylindrical, (6–)9–20(–26) × (2.5–)3–5(–6) µm, straight or slightly curved, rounded or truncated at both ends, slightly constricted at the septa, disposed in long branched or unbranched chains, secession schizolytic (Figure 12F–H). *Sexual state* (produced in vivo on leaves of *Pinus silvestris*, *P. maritima,* and *P. nigra*) consisting of superficial, scattered to gregarious, dark brown to black, carbonaceous thyriothecia-like ascomata, 100–200 μm high × 78–94 μm diameter ($\bar{X}$ = 85 × 146 μm; *n* = 5), opening by a longitudinal or Y-shaped sunken slit; peridium 10–23.7 μm wide, thinner at the base, comprising of condensed hyphae; hamathecium comprising numerous, 1.1–1.9 μm wide, filiform, flexuous pseudoparaphyses; *asci* 8-spored, bitunicate, cylindrical-clavate to clavate, 37–43 × 7.9–11.5 μm ($\bar{X}$ = 41.3 × 9.6 μm; *n* = 10), with a short, broad pedicel, apically rounded, with a distinct ocular chamber; *ascospores* 2–3-seriate, partially overlapping, hyaline, smooth-walled to verrucose, ellipsoid to fusiform or ovoid with rounded ends, laterally compressed, 10–12 × 4.5–6 μm ($\bar{X}$ = 10.8 × 5 μm; *n* = 5), slightly constricted at the septum, with a mucilaginous appendage at each end.

*Culture characteristics*—(After 14 days at 25 °C) Colonies on MEA 2% 3–5 mm diameter, umbonate, circular, entire, tough and mostly bright, margins lobulate and slightly filamentous, floccose, ivy-green (1F3) to olive (2F4) and margins dark-grey (1F1); reverse dark-gray (1F1) (Figure 12A). Colonies on OA 5–6 mm diameter, circular, flat with a mound at the center and opaque, margins entire and regular, dark-green (30F4), velvety mostly at the central area with celadon green (30D3) hyphae, and dull-green (30E4) margins; reverse dark-green (30F4) with dull-green (30E4) margins (Figure 12B). Colonies on PCA 3.5–4 mm diameter, convex to pulvinate, wrinkled, circular, entire, tough, and mostly bright, margins lobulate and slightly filamentous, sparse velvety, greenish-grey (30F2) and with dull-green (30E3) hyphae; reverse bronze-green (30F3) (Figure 12C). Colonies on PDA 4.5–6 mm diameter, convex, and wrinkled, circular, entire, tough and opaque, margins entire and regular, velvety, olive (1E3) to olive-brown (4E5) at the center, with spherical, black (6F3), slightly velvety, 27–50 × 23–43 stromata; reverse dark-gray (1F1) and margins olive (1E5) (Figure 12D,E).

*Specimen examined*—France, Trédarzec, on needles of *Pinus insignis* (Pineaceae), Coll. Desmaziere (CBS 174.90).

*Diagnosis*—The genus currently contains five species, *N. abietis* (the type species), *N. castaneae*, *N. germanicum*, *N. microsporus,* and *N. spinulosum*. *Neocatenulostroma pinorum* presents most of the traits of the genus, showing thallic conidiogenesis similar to *N. abietis* and *N. spinulosum*, but differs in the conidial size: 8–24 × 5–7 in *N. abietis*, and (5–)7–20(–22) × (2.5–)3–4(–5.5) µm in *N. spinulosum*. The colonies form pseudostromata on TWA with needles of *Pinus* sp. after four weeks at 25 °C, composed of quite compact hyphae, but not differentiated cells. *N. pinorum* is the only known species of the genus to produce a sexual morph in vivo.

*Notes*—Based on a BLAST search of the NCBI GenBank nucleotide database, the closest hit using the **ITS** sequence was *N. abietis* (strain CPC 14996, GenBank FJ372387.1; identities = 511/512 (99.80%) gaps 0/512 (0%)). Using the **LSU** sequence, it was *N. microsporus* (strain CBS 110890, GenBank EU019255.2; Identities = 1266/1289 (98.22%), gaps 23/1289 (1%)). For the ***rpb*2** sequence, it was *N. abietis* (strain CBS 459.93, GenBank OX431256; Identities = 793/833 (95.20%), gaps 0/833 (0%)). Our *rpb2* phylogenetic tree corroborates the placement of our strain as a species of the genus *Neocatenulostroma*. Due to the recent migration of the species to other genera [95], the genus *Aulographina* is currently invalidated.

**Figure 13:**
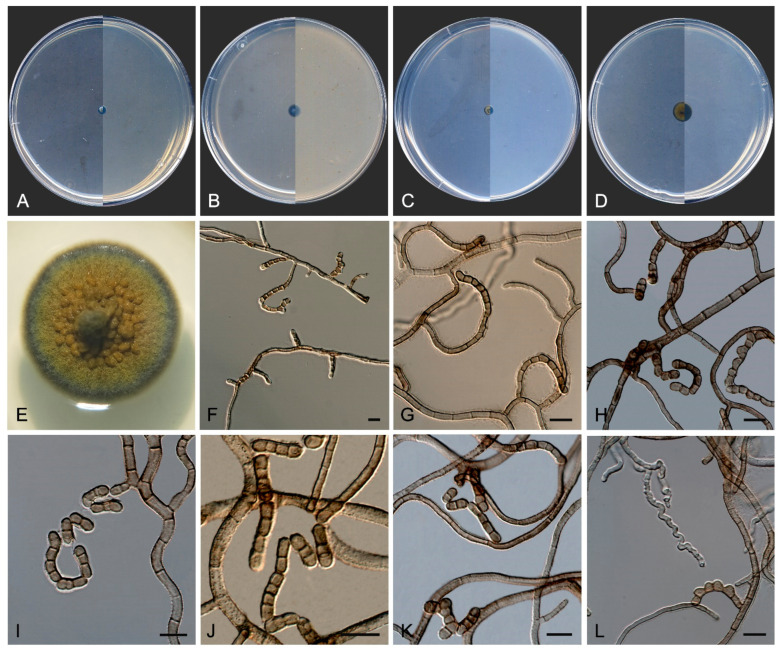
*Neocatenulostroma spinulosum* FMR 18793. (**A**–**D**) Colony on MEA, OA, PCA, PDA (after 2 wks at 25 ± 1 °C; surface, left; reverse, right). (**E**) Colony appearance on PDA. (**F**–**K**) Mycelium and fertile hyphae from which arthroconidia are released by schizolytic secession. (**L**) Asperulate conidia, 0–5 septa. DIC Nomarski. Scale bars = 10 μm.

*Neocatenulostroma spinulosum* Sastoque, Cano and Stchigel, sp. nov. Mycobank MB847923. Figure 13.

*Etymology*—From Latin *spinulosus*, having small spines, because of the ornamentation of the hyphae and conidia with age.

*Description*—On oatmeal agar after two weeks at 25 °C—*Mycelium* consisting of septate, branching, pale brown to brown, smooth- and thin-walled hyphae, 2–4.5 µm wide, from which arise laterally the fertile hyphae (Figure 13F–H,L). *Arthroconidia* 0–5-septate, pale brown to brown, smooth to asperulate, thick-walled, sub-cylindrical to cylindrical, (5–)7–20(–22) × (2.5–)3–4(–5.5) µm, straight or slightly curved, rounded or truncated at the ends, slightly constricted at the septa, disposed in long unbranched or branched chains, secession schizolytic (Figure 13I–K). *Sexual morph* not observed.

*Culture characteristics*—(After 14 days at 25 °C) Colonies on MEA 8 mm diameter, umbonate and acuminate, circular, entire, restricted, tough and bright, margins entire and flat, black (6F3), with sparse groups of olive (1F5) aerial hyphae, margins olive (1F4); reverse dark-gray (1F1) and margins olive (1F4) (Figure 13A). Colonies on OA 6 mm diameter, circular, flat with a mound in the center and bright, margins entire, bronze-green (30F3) with sparse greenish-grey (30E3) aerial mycelium in the central area, and grass-green (30E7) margins; reverse bronze green (30F3) with olive (30E7) margins (Figure 13B). Colonies on PCA 8–9 mm diameter, pulvinate and wrinkled at the top, circular, entire, tough and mostly opaque, margins entire and submerged on the culture medium, velvety with yellow-green (3F3) hyphae, with patches bright and dark-grey (1F1) in the central area and margins dark-green (1F3) without aerial hyphae; reverse dark-gray (1F1), margins dark-green (1F3) (Figure 13C). Colonies on PDA 7.5–9 mm diameter, circular, entire, tough, opaque, crater-shaped with smooth, bright and spherical bumps at the center, margins entire and slightly buried on the culture medium, greyish-green (2E6) with velvety hyphae, bright and dark-grey (1F1) with olive-brown (4E5) bumps in the central area, margins olive-green (2F6) without aerial hyphae; reverse dark-gray (1F1) and margins olive-green (2F6) (Figure 13D,E). Minimum, optimum, and maximum temperature of growth: 5 °C, 20 °C and, 25 °C, respectively.

*Type*—Spain, Tarragona Province, Els Pallaresos, isolated from a darkened PVC pipe for pluvial drain at an industrial warehouse N 41°10′36.2″ E 1°16′12.4″, 11 November 2020, coll. J. F. Cano-Lira and A. M. Stchigel, isol. A. P. Sastoque (holotype CBS H-25262, ex-type FMR 18793 = CBS 150899; ITS, LSU, *rpb2,* and *tef1* sequences GenBank OX628944, OX628945, OX628946 and OX628947, respectively).

*Diagnosis*—*Neocatenulostroma spinulosum* differs from *N. germanicum*, its phylogenetically closest species, by the conidiogenesis, which is holoblastic in *N. germanicum*, as well as in the size and the septation of the conidia, which are (8–)10–15(–20) × 4–5(–6) μm in size and obliquely septate in *N. germanicum* [96].

*Notes*—Based on a mega BLAST search of NCBIs GenBank nucleotide database, the closest hit using the **ITS** sequence was *A. pinorum* (strain CBS 302.71 (Type material), GenBank GU214622.1; identities = 468/470 (99.57%) gaps 0/470 (0%)). Using the **LSU** sequence, it was *N. abietis* (strain CBS 290.90, GenBank MH873896.1; Identities = 473/471 (98.34%), gaps 0/481 (0%)). For the ***rpb*2** sequence, it was *N. microsporum* (strain CBS 101951, GenBank OX431256; Identities = 830/873 (95.07%), gaps 2/873 (0%)). Regarding the ***tef*1** sequence, it was *N. abietis* (isolate AFTOL-ID1789, GenBank DQ677933.1; identities = 774/791 (97.85%) gaps 0/791 (0%)).

**Figure 14:**
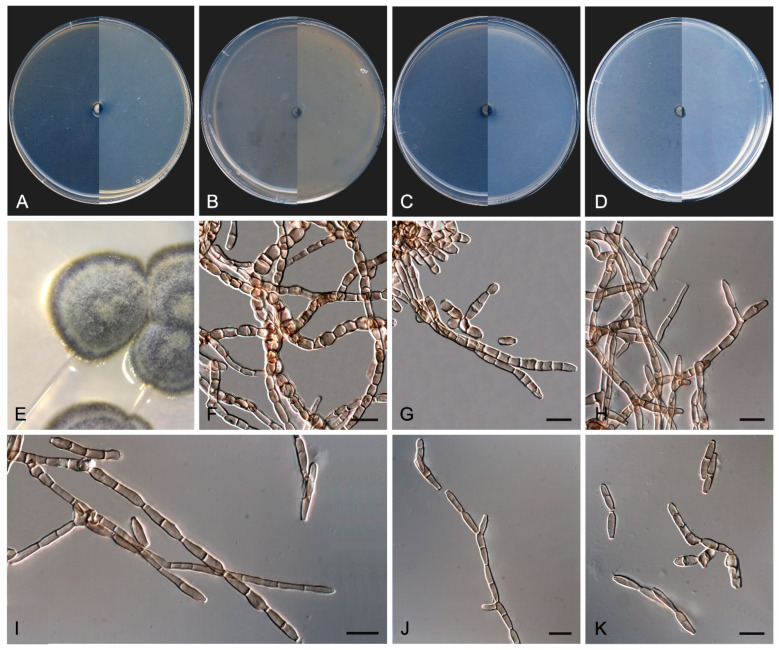
*Neodevriesia longicatenispora* FMR 18825. (**A**–**D**) Colony on MEA, OA, PCA, PDA (after 12 wks at 25 ± 1 °C; surface, left; reverse, right). (**E**) Colony appearance on PDA at 25 ± 1 °C. (**F**) Torulose hyphae. (**G**–**I**) Conidiophores and conidia. (**J**) Ramoconidia. (**K**) Free conidia or in chains. DIC Nomarski. Scale bars = 10 μm.

*Neodevriesia longicatenispora* Sastoque, Stchigel and Cano, sp. nov. Mycobank MB 847920. Figure 14.

*Etymology*. From Latin *longus*-, long, -*catena-*, chain, and -*sporis*, spores, because of the production of conidia in long chains.

*Classification*—Neodevriesiaceae, Capnodiales, Dothideomycetes, Pezizomycotina, Ascomycota.

*Description*—On oatmeal agar after twelve weeks at 25 °C—*Mycelium* consisting of pale brown to olivaceous brown, smooth- and thick-walled, septate, branching, isodiametric to slightly torulouse hyphae, 2–3 µm wide (Figure 14F). *Conidiophores* arising from the hyphae, mononematous, olivaceous brown, smooth- and thick-walled, straight to flexuous, unbranched, 1–12-septate, sub-cylindrical, 10–80 × 2–4 µm (Figure 14G–I). *Conidiogenous* cells sympodially proliferating, integrated or terminal, septate or non-septate, brown, sub-cylindrical, 5–14 × 3–4 µm, with flattened scars,1–3 µm wide, darkened along the rim, neither thickened nor refractive. *Ramoconidia* 0–1(–2)-septate, olivaceous brown, smooth- and thick-walled, constricted at both ends and slightly constricted at septa, sub-cylindrical, 6–17 × 2.5–4 µm (Figure 14J). *Conidia* 0–1-septate, olivaceous-brown to light brown, smooth- and thick-walled, disposed in persistent, long, branching chains, tapering towards both ends, sub-cylindrical to narrowly fusoid, 6–18 × 2–4 µm, slightly truncate at septum level, scars flattened, somewhat darkened and thickened, 1–3 µm wide (Figure 14K). *Chlamydospores* absent.

*Culture characteristics*—(After 14 days at 25 °C, Figure 14A–E) Colonies reaching 2 mm diameter on MEA and PCA, 3 mm on OA, and 3.5 mm on PDA, circular, entire, and regular margins. Pulvinate on MEA, OA, and PCA, crater-like on PDA, compact, dry, and velvety due to the aerial mycelium. Greyish green (29E5) on the surface, with white aerial mycelium (29A1) on MEA, OA, and PDA, but dull green (30E4) with bronze margins (30F3) on PCA and dark grey (1F1) with margins dark green to greyish green (29F5-30E5) reverse on all culture media tested. Minimum, optimum, and maximum temperature of growth: 15 °C, 25 °C, and 25 °C, respectively.

*Type*—Spain, Tarragona Province, Els Pallaresos, isolated from a blackened metal railing of an industrial warehouse N 41°10′28.6″ E 1°16′40.3″, 11 November 2020, coll. J. F. Cano-Lira and A. M. Stchigel, isol. A. P. Sastoque (holotype CBS H-25246, ex-type FMR 18825 = CBS 149963; ITS, LSU, *tub2* and *rpb2* sequences GenBank OX342400, OX342401, OX342226 and OX342225).

*Diagnosis*—*Neodevriesia longicatenispora* differs from *N. stirlingiae*, its phylogenetic closest species, by the size of conidiophores, conidiogenous cells, scars, ramoconidia, conidia, and hila, which are 10–50 × 4–5 μm, 8–15 × 3–4 μm, 1–2 μm diameter, 15–30 × 4–5 μm, (7–)12–16(–20) × (3–)4(–5) μm and 1–2 μm diameter, respectively, in *N. stirlingiae*. The number of septa in ramoconidia and conidia is also different among them, being 1–3 septate and 0–3 septate in *N. stirlingiae*, respectively. Moreover, *N. stirlingiae* does not form long chains of conidia and produces chlamydospores (absent in *N. longicatenispora*) [97].

*Notes*—Based on a mega BLAST search of NCBIs GenBank nucleotide database, the closest hit using the **ITS** sequence was *N. fraserae* (strain CBS 128217 (Type material), GenBank NR_144961.1; identities = 521/527 (98.86%) gaps 1/527 (0%)). Using the **LSU** sequence, it was *N. stirlingiae* (strain CPC 19948, GenBank NG_042755.1; Identities = 848/854 (99.30%), gaps 0/854 (0%)) and *N. fraserae* CBS 128217 (GenBank OX346373; Identities = 575/583 (98.63%), gaps 0/583 (0%)). For the ***tub*2** sequence, it was *N. fraserae* (strain CBS 128217, GenBank OX346373; Identities = 317/340 (93.24%), gaps 3/340 (0%)) and *N. stirlingiae* (strain CPC 19948, GenBank OX346410; Identities = 386/428 (90.19%), gaps 7/428 (1%)). Regarding the ***rpb*2** sequence, it was *N. fraserae* (strain CBS 128217, GenBank OX346372; Identities = 777/830 (93.61%), gaps 0/830 (0%)) and *N. stirlingiae* (strain CPC 19948, GenBank OX346315; Identities = 685/736 (93.07%), gaps 0/736 (0%)).

**Figure 15:**
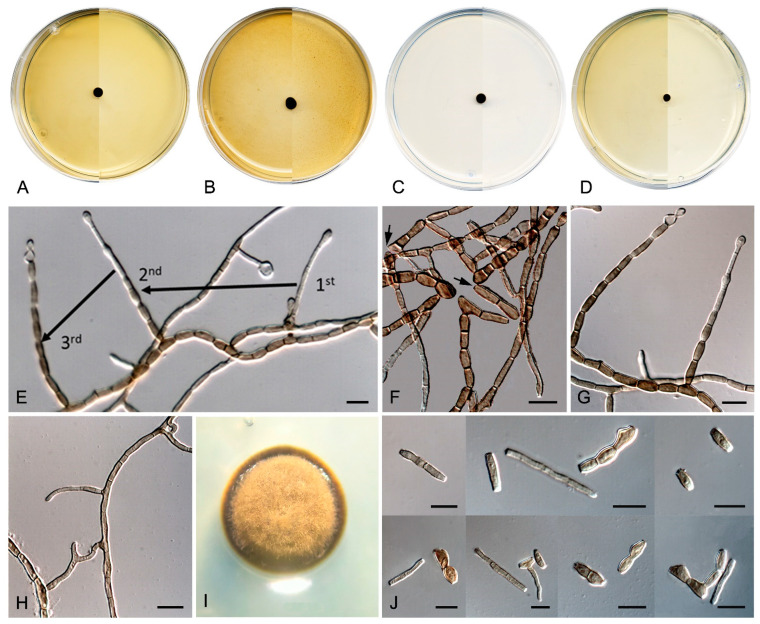
*Paradevriesia holothallica* FMR 18795. (**A**–**D**) Colony on MEA, OA, PCA, PDA (after 2 wks at 25 ± 1 °C; surface, left; reverse, right). (**E**) Conidiogenesis: hyphae become torulose and then disarticulate with schizolitic secession. (**F**) Disarticulation of conidia with schizolytic secession (black arrows). (**G**) Hyphae with swollen terminal cells. (**H**) Anastomosis. (**I**) Colony appearance on PDA at 25 ± 1 °C. (**J**) Holothallic conidia. DIC Nomarski. Scale bars = 10 μm.

*Paradevriesia holothallica* Sastoque, Cano and Stchigel, sp. nov. MycoBank MB 842105. Figure 15.

*Etymology*. From Greek *όλος*-, whole, and -*θαλλός*, sprout, in reference to the sort of the conidial ontogeny (holothallic).

*Classification*—Paradevriesiaceae, Capnodiales, Dothideomycetes, Pezizomycotina, Ascomycota.

*Description*—On potato dextrose agar after two weeks at 25 °C—*Mycelium* composed of septate, brown, guttulate, smooth to slightly verrucose, moderately thick-walled, branching hyphae, 1.5–3 μm wide; anastomosis frequently present and terminal part of the hyphae may swell (Figure 15G,H). *Conidiophores* absent. *Conidia* holothallic, 0–2 septate, brown, moderately thick-walled, smooth to slightly verruculose, guttulate, mostly prismatic, sub-cylindrical or barrel-shaped, occasionally T-shaped or ellipsoidal, almost flattened at both ends and without scars, 5–15 × 3–5 μm (Figure 15J), formed by remodeling and disarticulation of pre-existing hyphae sections by schizolytic secession (Figure 15E,F). After 12 wks on PDA at 25 °C, hyphae become moniliform, and the conidia are mostly barrel-shaped to ellipsoidal.

*Culture characteristics*—(After 14 days at 25 °C, Figure 15A–D,I) Colonies reaching 5 mm diameter on MEA, 6 mm on OA and PCA, and 3 mm on PDA, circular with wide, glistening, and regular margins. Flat to raise on OA and PCA, convex to pulvinate on MEA and PDA, compact, granulose, dry, velvety due to the aerial mycelium on PDA, and slightly velvety and glistening on MEA, OA, and PCA. Greenish black (30F4) on the surface, with black margins (6F3) and a black (6F3) reverse on all culture media tested. Minimum, optimum, and maximum temperature of growth: 15 °C, 25 °C, and 30 °C, respectively.

*Type*—SPAIN, Tarragona Province, Els Pallaresos, isolated from the darkened surface of a wall surrounding a garden N 41°10′31.3″ E 1°16′39.2″, 20 November 2020, coll. J. F. Cano-Lira and A. M. Stchigel, isol. A. P. Sastoque (holotype CBS H-24939, ex-type FMR 18795 = CBS 149012; ITS, LSU, and *rpb2* sequences GenBank OX031242, OX031243 and OX031309).

*Diagnosis*—*Paradevriesia holothallica* presents most of the traits of the genus but lacks conidiophores, the conidia have a holothallic ontogeny, the *hila* are absent, size and shape of conidia are very variable and have a schizolytic secession.

*Notes*—Based on a mega BLAST search of NCBIs GenBank nucleotide database, the closest hit using the **ITS** sequence was *P. compacta* (strain CBS 118294 (Type material), GenBank NR_144955.1; identities = 422/458 (92.14%) gaps 8/458 (1%)); using the **LSU** sequence it was *P. compacta* (strain CBS 118294, GenBank NG_059089.1; Identities = 760/784 (96.94%), gaps 2/784 (0%)) and for ***rpb2*** it was *Capnodiales* sp. (strain CBS 118294, GenBank GU371751.1; identities = 350/397 (88.16%) gaps 0/397 (0%)).

### 3.4. Physiology

The results of the carbon sources assimilation test for our strains are given in Table S3. *Exophiala* strains, *P. kinklidomatophilus*, *K. perfecta,* and *K. epidermidis* displayed the lowest spectrum of organic molecules assimilated. The strains of *E. xenobiotica* (FMR 19066 and CBS 118157) presented the same assimilation profile, while FMR 18810 was quite different. Among all *Exophiala* strains tested, *E. multiformis* showed the broadest range of carbon source assimilation. *Aureobasidium pullulans* and *A. microstrictum* were the species that showed greater differences than other *Aureobasidium* spp. in the assimilation pattern of organic compounds.

The results of the nitrogen sources assimilation test, fermentation, and production of acid from glucose, urease, and DNase for our strains are shown in Table S4. Most of the species assimilated all nitrogen sources tested, except *P. kinklidomatophilus* and *K. perfecta* which were not able to assimilate any of them; the assimilation pattern of *C. pleiosporus* (FMR 18827) was different from that observed in the other strains tested, assimilating only six of the eleven nitrogen sources used. The strains *A. pullulans*, *A. microstictum*, and *E. xenobiotica* (CBS 118157 and FMR 19066) were osmotolerant, whereas *C. pleiosporus* was weak. *K. epidermidis* and *E. xenobiotica* (CBS 118157 and FMR 19066) were tolerant to cycloheximide, and *P. kinklidomatophilus*, *K. perfecta,* and *C. pleiosporus* were negative for urease. All strains were negative for DNase. *A. pullulans*, *A. microstictum*, and *C. pleiosporus* produced acid from glucose. Notably, *P. kinklidomatophilus* and *K. perfecta* (FMR 18715) did not ferment any of the tested compounds, while *K. epidermidis* fermented almost all of them (except for galactose, lactose, and inulin). The strains belonging to the genera *Aureobasidium* and *Exophiala* showed a good fermentation capacity. Tolerance to NaCl was variable, *P. kinklidomatophilus*, *K. perfecta*, *E. caementiphila*, and *A. microstictum* were non-tolerant, while for MgCl_2_ and CaCl_2_ only *P. kinklidomatophilus* and *K. perfecta* were a little tolerant or intolerant (Table S5). Practically all strains grew from 5 °C to 30 °C, and between pH 3 and 11, but the strains *K. perfecta* and *E. xenobiotica* (FMR 19066) were able to grow in up to pH 12 (Table S5). The production of gelatinase was positive for *P. kinklidomatophilus*, *A. pullulans*, *C. pleiosporus,* and *A. microstictum* (Table S5).

## 4. Discussion

In our study of RIF involved in the alteration (darkening) of the surfaces of various urban buildings in different localities of the Province of Tarragona (Spain), 41 genera of fungi were found. Among them, *Alternaria*, *Cladosporium*, and *Penicillium* (present in all localities), and *Aspergillus*, *Aureobasidium*, *Beauveria*, *Curvularia*, *Epicoccum*, *Fusarium,* and *Trichoderma* (present only in Els Pallaresos and Reus) have been previously described as allergenic to humans [11,16,22,98,99]. Within these genera, 64 species have been identified, including *Alternaria infectoria*, *Aureobasidium pullulans*, *Didymella glomerata*, *Didymella microchlamydospora*, *Exophiala xenobiotica*, *Knufia epidermdis*, *Neoscytalidium dimidiatum*, and *Stemphylium vesicarium* (all found in Els Pallaresos, Montbrió del Camp and Reus), which are considered opportunistic pathogens for humans (Table S1) [27,36,42,100,101,102,103,104].

In addition, a new genus and six new species have been identified, all from darkened surfaces of the exterior walls of various buildings in the village of Els Pallaresos. *Coccodomyces pleiosporus* (Figure 8) was isolated from a metal fence and found to produce holoblastic conidia, cells with meristematic growth, and yeast-like cells. *Exophiala caementiphila* (FMR 18977) (Figure 9) was isolated from concrete and *E. multiformis* (FMR 18809) (Figure 10) from a metal fence; both species can be easily distinguished morphologically, the former produces abundant yeast-like cells while the latter is predominantly mycelial. *Neodevriesia longicatenispora*, isolated from the blackened metal railing of an industrial warehouse, produced cladosporium-like conidiophores (Figure 14), and *Paradevriesia holothallica*, isolated from the darkened surface of a wall surrounding a garden, showed holothallic conidiogenesis (Figure 15), making it the only species in the genus with this type of conidiogenesis. In addition, *Neocatenulostroma spinulosum* (Figure 13), the only fungus isolated from a PVC pipe, exhibits thallic–arthric conidiogenesis. This prompted us to review the conidia of other species [35,86] and to modify the description of the genus.

It was difficult to establish whether the meteorological conditions had a direct impact on the distribution and diversity of RIF in the sampled areas because no significant differences were found among the fungal communities isolated (Table 1). However, the fungal taxa we found were comparable to those reported in previous studies with similar meteorological data [5,7,8,10,21,39,48]. In addition, some factors that can favor the establishment and development of a rich RIF community are the presence of different plants as well as the composition of the soils surrounding the rocky substrates [1,4,8,11,26,105]. The local vegetation in our study consisted of Mediterranean crops, pine forests (Els Pallaresos), as well as ornamental plants (in Reus). This can influence in two ways, either because many RIF species have a life cycle linked to plants, animals, and humans, thus enabling them to reach these rocky substrates and colonize these niches successfully, or because nearby plants can contribute with decomposing materials (leaves, seeds, pollen, exudates, etc.) to enrich the rock surfaces with different types of compounds necessary for the germination of fungal propagules and the growth of RIF. This influence could be confirmed both by the species that have been considered as the first reports mentioned above, some of them better known as plant-associated, and by the frequency at which the plant-associated fungi have been found growing on rocky substrates [7,15]. On the other hand, the soil that surrounds them will also influence the fungal species that might reach the rocky substrates, i.e., by spreading the spores through the air [8,15,22,41]. In this sense, a substantial number of the strains isolated from all sampled sites (Figure 1) belonged to typical soil-borne fungal genera (i.e., *Aspergillus*, *Fusarium*, *Mucor*, and *Penicillium*,) or to fungi frequently observed in the soil (*Alternaria* and *Cladosporium*). Therefore, the influence of these factors could help explain the differences in RIF communities as well as their abundance among the points sampled in this study.

Regarding substrates, RIF can grow not only on different types of rocks (marble, stone, concrete, granite, sandstone, limestone, basalt, or travertine) but also on the surface of various hard materials, including glass, plastic, roof tiles, solar panels, steamers, humidifiers, and concrete dishwashers [2,4,6,9,21,26,39], as observed in our study (Table 2). These findings confirm the great flexibility and adaptability of RIF to different sorts of materials, suggesting that the chemical composition of the substrates has a minimal effect on fungal metabolism because, perhaps, the greatest part of its source of nutrients is external [8]. On the other hand, a potential positive association has been suggested between substrates with a high capacity for water retention and a diverse RIF fungal community [106]. As in previous reports [26,33,107], we found *A. pullulans* and *E. xenobiotica* on metallic structures, but also *P. brasiliense* and *Con. Leucoplaca*, as well as some of the new taxa, *C. pleiosporus*, *E. multiformis*, *N. longicatenulospora*, and *P. kinklidomatophilus* [90], which represent new reports for this material. On the other hand, only *N. spinulosum* was found on a PVC pipe, indicating its particular ability to colonize and develop on this polymer substrate, probably because *N. spinulosum* is an extremely oligotrophic fungus. The limited amount of nutrients in these materials would favor the development of RIF, with an oligotrophic metabolism, compared to cosmopolitan microorganisms, whose development is highly dependent on the nutrients available in such substrates [8].

The physiological characteristics of our tested RIF strains do not allow them to be considered as strictly extremophilic organisms (e.g., the tested strains do not show optimal growth at “high” or “low” temperatures, nor at a “low” humidity or “extreme pH”), as one might expect from microorganisms adapted to colonize the surface of various types of building materials whose chemical composition, in addition to their exposure to unfavorable weather conditions, makes them uninhabitable for most microorganisms.

## 5. Conclusions

Our results showed a high biodiversity of RIF in different sites and building materials sampled in the Province of Tarragona. Although this was comparable to the previous studies in urban areas of Europe, some of the RIF we found were first reports and also new species. The urban and industrial areas of the village of Els Pallaresos had the highest RIF biodiversity, followed by Reus. A large number of representatives of the order Pleosporales were found at all sampling sites, but representatives of the orders Capnodiales, Chaetothyriales, and Dothideales were also found. The present study also allowed us to discover a new genus (*Coccodomyces*) and six new species (*Coccodomyces pleiosporus*, *Exophiala caementiphila*, *Exophiala multiformis*, *Neocatenulostroma spinulosum*, *Neodevriesia longicatenispora*, and *Paradevriesia holothallica*) as well as to determine the correct phylogenetic placement of *Aulographina pinorum* (transferred to the genus *Neocatenulostroma*). Regarding the physiology of the RIF species, all tested strains exhibited growth within the temperature range of 5 to 30 °C and at pH levels between 3 and 11. However, osmotolerance was observed only in strains with the potential characteristics of extremophiles. We had expected that most strains would have a maximum growth temperature above 35 °C, considering their prolonged exposure to high temperatures in late spring and summer in the Mediterranean areas studied, but no thermophilic or highly thermotolerant fungi were found. Likewise, no xerophilic species of RIF were found. The resistance of the isolated RIF species to desiccation and temperature shocks must, therefore, be attributed to a thick melanized cell wall, the extracellular production of melanin, and the formation of biofilms [4,34].

## Figures and Tables

**Table 1:** Main climatological data for the sampling sites ^1^.

Parameters	Calafell	Els Pallaresos	Montbrió del Camp	Reus
Lineal distance to the shore (km)	0	5.7	7.4	9.0
Average annual temperature (°C)	15.9	16	15.6	15.0
Minimum average annual temperature (°C)	8.4	8	7.7	6.3
Maximum average annual temperature (°C)	24.2	24	24.3	24
Average annual rainfall (mm)	602	513	550	525
Relative humidity: lower value (%)	64.87	67.8	61.57	57.27
Relative humidity: highest value (%)	74.60	72.6	75.41	75.69
Prevailing wind direction	S/W	S/W	S/W	S/W

^1^ data from https://es.climate-data.org/ (accessed on 12 June 2023) and https://es.weatherspark.com/ (accessed on 12 June 2023) (S = South; W = Western).

**Table 2:** Sampling points, geographical location, and sort of sampled sources.

Town	Sample Name	Coordinates (UTM) *	Source
Calafell	C1	31T 79,177.10 61,802.90	Darkened concrete fence of a garden house
	C2	31T 79,134.60 61,766.60	Darkened concrete wall
	C3	31T 79,796.60 61,222.00	Darkened concrete wall
	C4	31T 78,841.90 61,117.50	Blackened block wall
	C5	31T 78,584.00 61,023.10	Darkened concrete wall
Montbrió del Camp	M1	31T 32,720.20 54,291.00	Darkened concrete wall
	M2	31T 32,658.70 54,252.30	Darkened brick wall
	M3	31T 32,414.20 54,091.30	Darkened concrete wall
	M4	31T 32,413.80 54,090.90	Darkened concrete wall
	M5	31T 32,378.60 54,109.80	Darkened clay wall
Els Pallaresos (Urban area)	P1	31T 54,937.40 59,758.10	Darkened concrete wall
	P2	31T 54,954.60 59,694.90	Darkened brick wall
	P3	31T 54,952.50 59,638.90	Darkened concrete wall
	P4	31T 55,523.50 59,652.30	Darkened concrete fence of a home garden
	P5	31T 55,545.50 59,571.40	Darkened metal railing of a small natural park
Els Pallaresos (Industrial area)	S1	31T 54,978.50 59,782.70	Darkened concrete wall
	S2	31T 54,945.50 59,754.60	Darkened metal fence near a tree
	S3	31T 54,916.20 59,764.70	Blackened cement blocks wall
	S4	31T 54,899.30 59,817.50	Darkened PVC pipe for pluvial drain
	S5	31T 54,946.80 59,861.60	Darkened concrete wall
Reus	R1	31T 49,684.80 57,714.70	Darkened concrete wall
	R2	31T 49,916.90 57,650.50	Darkened concrete wall
	R3	31T 42,102.90 57,713.20	Darkened concrete wall
	R4	31T 46,377.40 57,475.40	Darkened brick wall
	R5	31T 41,270.60 58,092.00	Blackened block wall

* UTM = Universal Transverse Mercator.

**Table 3:** List of primers and annealing temperatures used for amplification of gene targets.

Locus	Primer	Sequence (5′→3′) *	Orientation	Annealing Temperature (°C)	Reference
ITS/LSU	ITS5	GGAAGTAAAAGTCGTAACAAGG	Forward	53–55	[66]
	LR5	ATCCTGAGGGAAACTTC	Reverse	53–55	[67]
LSU	NL1	GCATATCAATAAGCGGAGGAAAAG	Forward	53–55	[68]
	NL4b	GGTCCGTGTTTCAAGACGG	Reverse	53–55	[68]
*rpb*2	fRpb2-5F	GGGGWGGAYCAGAAGAAG	Forward	55–60	[69]
	fRpb2-7R	CCCATRGCTTGYTTRCCCAT	Reverse	55–60	[69]
*tub*2	T10	ACGATAGGTTCACCTCCAGAC	Forward	55–57	[70]
	Bt2a	GGTAACCAAATCGGTGCTGCTTTC	Forward	55–57	[71]
	Bt2b	ACCCTCAGTGTAGTGACCCTTGGC	Reverse	55–57	[71]
*tef*1	EF-1H	ATGGGTAAGGARGACAAGAC	Forward	57	[72]
	EF-2T	GGAAGTACCAGTGATCATGTT	Reverse	57	[72]
	EF1-983F	GCYCCYGGHCAYCGTGAYTTYAT	Forward	57	[73]
	EF1-2218R	ATGACACCRACRGCRACRGTYTG	Reverse	57	[73]
	EF1-728F	CATCGAGAAGTTCGAGAAGG	Forward	57	[74]
	EF1-986R	TACTTGAAGGAACCCTTACC	Reverse	57	[74]

* H = A, C or T; R = A or G; W = A or T; Y = C or T.

## Data Availability

Data are contained within the article and Supplementary Materials.

## Supplementary Materials

The following supporting information can be downloaded at: https://www.mdpi.com/article/10.3390/jof10030170/s1, Table S1: Geographical origin and sources, and loci sequenced and accession numbers (GenBank/EMBL) for their nucleotide sequences of the fungi isolated in this study [108,109,110,111,112,113,114]. Table S2: Geographical origin and sources, loci and accession numbers of nucleotide sequences of fungal strains included in the phylogenetic analysis. Table S3: Carbon source assimilation; Table S4: Nitrogen source assimilation, osmotolerance, cycloheximide resistance, urease and DNAse production, acid from glucose and sugar fermentation for the fungal strains tested; Table S5: Halotolerance, thermotolerance, pH tolerance, and gelatinase production for the fungal strains tested.
