# Supplementary material for: Diversity of Rock-Inhabiting Fungi in Tarragona Province, Spain
Source: J Fungi (Basel). 2024 Feb 22;10(3):170. doi: 10.3390/jof10030170 (PMC10971471; doi:10.3390/jof10030170)
Supplement: Supplementary file 1 [file jof-10-00170-s001.zip › jof-2805531-supplementary.pdf]

Table S1. Geographical origin and sources, and *loci* sequenced and accession numbers (GenBank/EMBL) for their nucleotide sequences of the fungi isolated in this study.

Species	Strain	Culture collection	Country	Sample*/ Isolation source	GenBank accession numbers					Reference
					ITS	LSU	*rpb2*	*tub2*	*tef1*	
Acremonium domschii	FMR 19485	-	Spain	M4	**OX431181**	-	-	-	-	This study
Acrophialophiora sp.	-	-	Spain	C3	-	-	-	-	-	This study
Allophoma labilis	FMR 18605	-	Spain	M4	-	-	**OY101427**	-	-	This study
Allophoma labilis	FMR 18606	-	Spain	M5	-	-	**OY101428**	-	-	This study
Allophoma labilis	FMR 18613	-	Spain	P2	-	-	**OX345722**	-	-	This study
Allophoma labilis	FMR 18621	-	Spain	R3	-	-	**OX345725**	-	-	This study
Alternaria infectoria	FMR 18710	-	Spain	P2	**OX336879**	-	-	-	-	This study
Alternaria spp.	-	-	Spain	C1, C2, C4, C5, M1, M2, M3, M4, P1, P2, P3, P4, P5, R1, R2, R4, R5, S1, S3, S5	-	-	-	-	-	This study
Angustimassarina rosarum	FMR 18796	-	Spain	P4	-	-	-	-	**OX346378**	This study
Apiospora marii	FMR 18616	-	Spain	R1	**OX336876**	-	-	-	-	This study
Aplosporella sp.	-	-	Spain	R2	-	-	-	-	-	This study
Arthrinium spp.	-	-	Spain	C2, C5	-	-	-	-	-	This study
Aspergillus sect. Nidulata	-	-	Spain	P4	-	-	-	-	-	This study
Aspergillus sect. *Nigri*	-	-	Spain	R2, R3	-	-	-	-	-	This study
Aspergillus sect. *Versicolores*	-	-	Spain	C4	-	-	-	-	-	This study
Aspergillus sp.	-	-	Spain	S1	-	-	-	-	-	This study
Aureobasidium pullulans	FMR 18808	-	Spain	S2	**OX346306**	-	-	-	-	This study
Aureobasidium pullulans	FMR 19740	-	Spain	R3	**OX346314**	-	-	-	-	This study
Aureobasidium pullulans	FMR 19755	-	Spain	R1	-	-	**OX346389**	-	-	This study
Aureobasidium spp.	-	-	Spain	P4, S2, S3, S5	-	-	-	-	-	This study
Beauveria pseudobassiana	FMR 18975	-	Spain	S1	-	-	-	-	**OX346387**	This study
Beauveria sp.	-	-	Spain	S2	-	-	-	-	-	This study
Candida davisiana	FMR 19484	-	Spain	M4	**OX346398**	-	-	-	-	This study
Cladosporium spp.	-	-	Spain	C1, C2, C4, C5, M1, M2, M3, M4, M5, P1, P2, P3,	-	-	-	-	-	This study

				P4, P5, R1, R2, R3, R4, S1, S2, S3, S4, S5						
Clonostachys solani f. nigrovirens	FMR 19742	-	Spain	M4	**OX346399**	-	-	-	-	This study
Coccodomyces pleosporus	**FMR 18827**	**CBS 149014^{T}**	**Spain**	**S2**	**OW273979**	**OW370575**	-	-	-	This study
Coniochaeta leucoplaca	FMR 18813	-	Spain	P5	-	**OX346308**	-	-	-	This study
Coniosporium apollinis	FMR 18714	-	Spain	C5	**OX346304**	-	-	-	-	This study
Coniosporium uncinatum	FMR 18792	-	Spain	P2	-	-	**OX431254**	-	-	This study
Coniosporium uncinatum	FMR 18794	-	Spain	S1	-	-	**OX628948**	-	-	This study
Coniosporium uncinatum	FMR 19658	CBS 100219^{T}	France	Stone	-	-	-	-	-	[108]
Cosmospora lavitskiae	FMR 19486	-	Spain	R4	**OX346313**	-	-	-	-	This study
Curvularia sp.	-	-	Spain	S1	-	-	-	-	-	This study
Cystofilobasidium capitatum	FMR 19756	-	Spain	R3	**OX346400**	-	-	-	-	This study
Didymella glomerata	FMR 18789	-	Spain	M4	-	-	**OX346409**	-	-	This study
Didymella glomerata	FMR 18805	-	Spain	P2	-	-	**OX346380**	-	-	This study
Didymella microchlamydospora	FMR 18600	-	Spain	M1	-	-	**OY101425**	-	-	This study
Didymella microchlamydospora	FMR 18626	-	Spain	R4	-	-	**OX345728**	-	-	This study
Didymella pomorum	FMR 18814	-	Spain	M4	-	-	**OX346384**	-	-	This study
Didymella sp.	-	-	Spain	M3	-	-	-	-	-	This study
Dimorphoma saxea	FMR 18611	-	Spain	C5	-	-	**OX345720**	-	-	This study
Dimorphoma saxea	FMR 18612	-	Spain	P1	-	-	**OX345721**	-	-	This study
Dimorphoma saxea	FMR 18614	-	Spain	P3	-	-	**OX345723**	-	-	This study
Dimorphoma saxea	FMR 18617	-	Spain	R1	**OX336877**	-	-	-	-	This study
Dimorphoma saxea	FMR 18628	-	Spain	R5	**OX336878**	-	-	-	-	This study
Dothiora mahoniae	FMR 19656	CBS 264.92^{T}	USA	*Mahonia repens*	MH862357	**OX346371**	-	-	-	[109]
Dothiorella sarmentorum	FMR 18713	-	Spain	R1	**OX336880**	-	-	-	-	This study
Epicoccum spp.	-	-	Spain	C4, P1, R1, R5, S3, S5	-	-	-	-	-	This study
Exophiala caementiphila	**FMR 18977**		**Spain**	**S3**	**OX380503**	**OX380504**	**-**	**OX380502**	**OX380501**	This study
Exophiala multiformis	**FMR 18809**	**CBS 149013^{T}**	**Spain**	**S2**	**OU624179**	**OU624180**	**-**	**OU624443**	**OU624442**	This study
Exophiala xenobiotica	FMR 18810	-	Spain	S2	**OX346307**	-	-	-	-	This study
Exophiala xenobiotica	FMR 18979	-	Spain	P2	**OX346311**	-	-	-	-	This study
Exophiala xenobiotica	FMR 19066	-	Spain	P5	**OX346312**	-	-	-	-	This study
Exophiala xenobiotica	FMR 19661	CBS 118157^{T}	Venezuela	Oil sludge	NR_111203	-	-	-	-	[30]
Fusarium acuminatum	FMR 18812	-	Spain	S5	-	-	**OX346383**	-	-	This study

Fusarium babinda	FMR 18804	-	Spain	M4	-	-	-	-	**OX346379**	This study
Fusarium salinense	FMR 18620	-	Spain	R2	-	-	-	-	**OX345724**	This study
Fusarium spp.	-	-	Spain	M1, M3, M4, P1, R1, R2, R4, S5	-	-	-	-	-	This study
Gonatobotryum apiculatum	FMR 20101	CBS 182.68	Canada	Soil under *Pinus strobus*	**OX346374**	**OX346375**	-	-	-	[110]
Juxtiphoma yunnanensis	FMR 18815	-	Spain	P2	-	-	**OX346385**	-	-	This study
Aureobasidium microstictum	FMR 19067	-	Spain	S2	-	-	**OX346388**	-	-	This study
Knufia epidermidis	FMR 18978	-	Spain	S3	**OX346310**	-	-	-	-	This study
Knufia perfecta	FMR 18715	-	Spain	C5	**OX346305**	-	-	-	-	This study
Lithohypha guttulata	FMR 18791	-	Spain	P2	**OX431178**	**OX431179**	-	**OX431253**	**OX431252**	This study
Lithophila guttulata	FMR 20100	CBS 139723^{T}	Italy	Cortile della Pigna, Vatican Museum	-	-	-	**OX346320**	**OX346319**	[7]
Mucor fragilis	FMR 18770	-	Spain	R3	**OX336881**	-	-	-	-	This study
Mucor sect. *Mucido*	-	-	Spain	R3	-	-	-	-	-	This study
Neocatenulostroma abietis	FMR 19657	CBS 459.93^{T}	Germany	*Abies* sp.	-	MH874081	**OX431256**	-	-	[111]
Neocatenulostroma germanicum	FMR 19655	CBS 539.88^{T}	Germany	Stone	-	MH873835	**OX431255**	-	-	[111]
Neocatenulostroma microsporum	FMR 19659	CBS 101951^{T}	South Africa	*Protea cynaroides*, living leaf	-	KF901814	**OX431256**	-	-	[111]
Neocatenulostroma pinorum	FMR 19653	CBS 174.90^{T}	France	*Pinus insignis*, needles	-	GU301802	GU371737	-	-	[112]
Neocatenulostroma spinulosum	**FMR 18793**		**Spain**	**S4**	**OX628944**	**OX628945**	**OX628946**	-	**OX628947**	This study
Neodevriesia fraserae	FMR 19662	CBS 128217^{T}	Australia	*Melaleuca* sp., leaves	**OX346372**	**OX346373**	**OX346372**	**OX346373**	-	[113]
Neodevriesia longicatenulospora	**FMR 18825**		**Spain**	**P5**	**OX342400**	**OX342401**	**OX342225**	**OX342226**	-	This study
Neodevriesia stirlingiae	FMR 19654	CBS 133581^{T}	Australia	*Stirlingia latifolia*, leaves	**OX346369**	NG_042755	**OX346315**	**OX346410**	-	[114]
Neodidymelliopsis faroknejadii	FMR 18601	-	Spain	M2	-	-	**OY101426**	-	-	This study
Neoscytalidium dimidiatum	FMR 18624	-	Spain	R4	-	-	**OX345726**	-	-	This study
Nothophoma quercina	FMR 18625	-	Spain	R4	-	-	**OX345727**	-	-	This study
Paraconiothyrium brasiliense	FMR 18712	-	Spain	P5	-	-	**OX345732**	-	-	This study
Paradevriesia holothallica	**FMR 18795**	**CBS 149012^{T}**	**Spain**	**P4**	**OX031242**	**OX031243**	**OX031309**	-	-	This study

Paraphoma fimeti	FMR 18816	-	Spain	S5	-	-	**OX346386**	-	-	This study
Penicillium brevicompactum	FMR 18741	-	Spain	R3	-	-	-	**OX345733**	-	This study
Penicillium brevicompactum	FMR 18811	-	Spain	S5	-	-	-	**OX346382**	-	This study
Penicillium hordei	FMR 18806	-	Spain	S1	-	-	-	**OX346381**	-	This study
Penicillium sect. *Aspergilloides*	-	-	Spain	R2, R3, R5, S5,	-	-	-	-	-	This study
Penicillium sect. *Furcatum*	-	-	Spain	C2, P1, P5, R3, S1	-	-	-	-	-	This study
Penicillium spp.	-	-	Spain	C2, M3, P4, R3, R5, S1, S3, S4	-	-	-	-	-	This study
Phaeococcomyces kinklidomatophilus	FMR 18615	CBS 147696[T]	Spain	P5	HG995431	HG995460	-	-	-	[90]
Pseudoseptoria obscura	FMR 18976	-	Spain	S1	**OX346309**	-	-	-	-	This study
Sordaria clematidis	FMR 18629	-	Spain	R5	-	-	-	-	**OX345729**	This study
Stemphylium vesicarium	FMR 18655	-	Spain	P1	-	-	**OX345731**	-	-	This study
Talaromyces ramulosus	FMR 18654	-	Spain	C4	-	-	-	**OX345730**	-	This study
Talaromyces sp.	-	-	Spain	C3	-	-	-	-	-	This study
Thyridium vestitum	FMR 19738	-	Spain	R1	**OX431181**	-	-	-	-	This study
Trichoderma sp.	-	-	Spain	S4	-	-	-	-	-	This study

*See Table 1. [T], type strain. New species are **in bold**. Sequences obtained in this study are **in bold**. CBS, CBS-KNAW Westerdijk Fungal Biodiversity Institute (Utrecht, Netherlands). FMR, Facultat de Medicina (Reus, Spain).

References

7. Isola D, Zucconi L, Onofri S, Caneva G, de Hoog GS, Selbmann L. Extremotolerant rock inhabiting black fungi from Italian monumental sites. Fungal Divers. 2015;76:75–96. https://doi.org/10.1007/s13225-015-0342-9

30. de Hoog GS, Zeng JS, Harrak MJ, Sutton DA. *Exophiala xenobiotica* sp. nov., an opportunistic black yeast inhabiting environments rich in hydrocarbons. Antonie Van Leeuwenhoek. 2006;90:257–68. https://doi.org/10.1007/s10482-006-9080-z

90. Crous PW, Osieck ER, Jurjević, Boers J, Van Iperen AL, Starink-Willemse M, et al. Fungal Planet description sheets: 1284-1382. Persoonia. 2021;47:178–374. https://doi.org/10.3767/persoonia.2021.47.06

108. De Leo F, Urzì C, de Hoog GS. Two *Coniosporium* species from rock surfaces. Stud Mycol [Internet]. 1999;1999:70–9. Available from: https://www.researchgate.net/publication/258925914_Two_Coniosporium_species_from_rock_surfaces

109. Crous PW, Wingfield MJ, Carnegie AJ, Lombard L, Roux J, Barreto RW, et al. Fungal Planet description sheets : 785 – 867. Persoonia. 2018;41:238–417. https://doi.org/10.3767/persoonia.2018.41.12

110. Hughes SJ. Conidiophores, conidia, and classification. Can J Bot. 1953;31:577–659. https://doi.org/https://doi.org/10.1139/b53-046

111. Quaedvlieg W, Binder M, Groenewald JZ, Summerell BA, Carnegie AJ, Burgess TI, et al. Introducing the consolidated species concept to resolve species in the Teratosphaeriaceae. Persoonia. 2014;33:1–40. https://doi.org/10.3767/003158514X681981

112. von Arx JA, Müller E. Über die neue Ascomycetengattung *Aulographina*. Sydowia [Internet]. 1960;14:330–3. Available from: https://www.zobodat.at/pdf/Sydowia_14_0330-0333.pdf

113. Wang MM, Shenoy BD, Li W, Cai L. Molecular phylogeny of *Neodevriesia*, with two new species and several new combinations. Mycologia [Internet]. 2017;109(6):965–74. Available from: https://doi.org/10.1080/00275514.2017.1415075

114. Crous PW, Schumacher RK, Wingfield MJ, Lombard L, Giraldo A, Christensen M, et al. Fungal systematics and evolution: FUSE 1. Sydowia. 2015;67(December 15):118. https://doi.org/10.12905/0380.sydowia67-2015-0081

Table S2: Geographical origin, sources, *loci* and accession numbers of nucleotide sequences of fungal strains included in the phylogenetic analysis.

Species	Culture collection	Country	Isolation source	GenBank accession numbers		
				ITS	LSU	*rpb2*
Amycosphaerella africana	CBS 116154^T	India	*Eucalyptus viminalis*, leaves	KF901700	KF902047	-
Aureobasidium pullulans	CBS 584.75^T	France	*Vitis vinifera*, fruit	-	NG_055734	-
Austroafricana associata	CBS 120730^T	Australia	*Corymbia henryii*, leaves	-	KF901824	-
Austroafricana parva	CBS 122892^T	Australia	*Eucalyptus globulus*, leaves	-	MH874775	-
Brunneosphaeria jonkershoekensis	CBS 130594	South Africa	*Protea repens*, leaves	NR_156244	NG_058654	-
Capronia mansonii	CBS 101.67^T	Sweden	*Populus tremula*	-	MH870591	-
Cladophora banthiana	CBS 100429	Unknown	Human, brain abscess	KF155212	MH877849	-
Cladophora banthiana	CBS 101158	Japan	Human, brain infection	AY857516	-	-
Cladophora carrionii	CBS 260.83	Uganda	Human, skin lesion	MH861582	MH873312	-
Devriesia staurophora	CBS 375.81	Colombia	Paramo soil	-	KF901963	-
Devriesia tardicrescens	CBS 128770^T	South Africa	*Phaenocoma prolifera*, leaf bracts	NR_137771	NG_059091	-
Dothidea sambuci	CBS 198.58	Switzerland	*Acer pseudoplatanus*	AY930109	AF382387	-
Dothiora maculans	CBS 301.76	Canada	*Populus tremuloides*, leaf litter	-	MH872751	-
Exophiala alcalophila	CBS 122256	Denmark	Human, toenail	JF747044	-	-
Exophiala alcalophila	CBS 521.82	Japan	Soil	JF747042	-	-
Exophiala alcalophila	CBS 520.82^T	Japan	Soil	NR_111624	NG_059189	-
Exophiala angulospora	CBS 482.92^T	Japan	Water from drinking well	NR_111625	NG_070601	-
Exophiala angulospora	CBS 146.93	Germany	*Tilia platyphylla*, wood	JF747053	-	-
Exophiala angulospora	CBS 122264	Denmark	Human, toenail	JF747052	-	-
Exophiala asiatica	CBS 122847^T	China	Human, wound infection of tonsils	NR_111332	-	-
Exophiala bergeri	CBS 353.52^T	Canada	Human, chromomycosis	NR_165997	NG_059199	-
Exophiala bergeri	CBS 526.76	USA	Human, subepidermal cyst	MH861000	-	-
Exophiala castellanii	CBS 158.58^T	Sri Lanka	Human	MH857734	NG_070513	-
Exophiala crusticola	CBS 119970^T	USA	Soil, biological soil crust sample	NR_159867	NG_059220	-
Exophiala dermatidis	CBS 109154	South Korea	Human, brain	AY857525	-	-
Exophiala dermatidis	CBS 686.92	Germany	Human	MF320155	-	-

Exophiala dermatidis	CBS 120473	USA	Human, brain	MF320159	-	-
Exophiala dermatidis	CBS 207.35^{T}	Japan	Human, subcutaneous phaeohyphomycosis	MH855649	NG_059225	-
Exophiala halophila	CBS 121512^{T}	USA	Human, skin axilary	NR_111628	-	-
Exophiala heteromorpha	CBS 633.69	Canada	Railway tie, wood of *Pinus banksiana*	AY857522	MH871160	-
Exophiala heteromorpha	CBS 116.97	USA	Soil polluted with petroleum	AY857521	-	-
Exophiala jeanselmei	CBS 677.76	England	Human, skin, abscess of foot	JN625228	-	-
Exophiala jeanselmei	CBS 507.90^{T}	Uruguay	Human, mycetoma	NR_111129	MH873915	-
Exophiala jeanselmei	CBS 528.76	Unknown	Human, skin, hand	AY857530	-	-
Exophiala lecanii-corni	CBS 102400	USA	Air supply passed through filter	AY857527	-	-
Exophiala lecanii-corni	CBS 232.39	Brazil	Human, chromomycosis	FJ974061	MH867492	-
Exophiala mesophila	CBS 402.95^{T}	Germany	Silicone seal, in shower room of hospital	NR_121461	KX712349	-
Exophiala mesophila	CBS 121507	USA	Human, hair	JF747120	-	-
Exophiala mesophila	CBS 836.95	Germany	Slime on floor outdoor swimming-pool	JF747112	-	-
Exophiala oligosperma	CBS 725.88^{T}	Germany	Human, tumour of sphenoidal cavity	NR_111134	NG_059201	-
Exophiala oligosperma	CBS 658.76	USA	Unknown	AY857532	-	-
Exophiala oligosperma	CBS 265.49	France	Honey	MH856519	MH868049	-
Exophiala phaeomuriphormis	CBS 131.88^{T}	Unknown	Human, phaeohyphomycosis	AJ244259	MH873815	-
Exophiala spinifera	CBS 356.83	Egypt	Unknown	AY156961	MH870977	-
Exophiala spinifera	CBS 425.92	Germany	Apple juice	AY156962	-	-
Exophiala spinifera	CBS 899.68^{T}	USA	Human, nasal granuloma	NR_111131	-	-
Exophiala xenobiotica	CBS 117753	USA	Human, leg wound	JN625227	-	-
Exophiala xenobiotica	CBS 117648	USA	Sclera	EF025407	-	-
Gonatobotryum apiculatum	CBS 182.68	Canada	Soil, under *Pinus strobus*	MH859103	MH870816	-
Helotium subcorticale	CBS 248.62	France	Unknown	-	MH869740	-
Hormonema macrosporum	CBS 536.94^{T}	Russia	*Rutilus rutilus*, gills	-	NG_064169	-
Hormonema merioides	CBS 906.85^{T}	Canada	*Pseudotsuga menziesii*, needle	MH861924	MH873614	-
Kabatina thujae	CBS 238.66^{T}	Germany	*Thuja occidentalis*, withering shoot	-	NG_064053	-
Neocatenulostroma castaneae	MFLUCC 17-2188^{T}	Italy	*Castanea sativa*	-	NG_081524	-
Neocatenulostroma pinorum	CBS 302.71	France	*Pinus maritima*	GU214622	GU214393	GU371766
Neodevriesia agapanthi	CBS 132689^{T}	South Africa	*Agapanthus africanus*, leaves	NR_111766	NG_042688	-

Neodevriesia bulbillosa	CBS 118285^{T}	Spain	Limestone rock, surface	NR_144953	KF310029	-
Neodevriesia cladophorae	CGMCC 3.17901^{T}	China	*Cladophora* sp., intertidal zone	KU578112	KU578114	-
Neodevriesia coccolobae	CBS 145064^{T}	Puerto Rico	*Coccoloba uvifera*, leaves	NR_161126	NG_066285	-
Neodevriesia grateloupiae	CGMCC 3.14281^{T}	China	*Grateloupia* sp., intertidal zone	KU578118	KU578120	-
Neodevriesia hilliana	CBS 123187^{T}	New Zeland	*Macrozamia communis*, leaves	NR_145098	MH874801	-
Neodevriesia imbrexigena	CAP1373^{T}	Portugal	Glazed decorative tiles in association with *Trebouxia* sp.	JX915747	JX915751	-
Neodevriesia knozdaviesii	CBS 122898^{T}	South Africa	*Protea* sp., leaves	MH863254	MH874778	-
Neodevriesia lagerstroemiae	CBS 125422^{T}	USA	*Lagerstroemia indica*	MH863701	KF902149	-
Neodevriesia metrosideri	CBS 145084^{T}	New Zeland	*Metrosideros* sp	NR_161141	NG_066296	-
Neodevriesia modesta	CBS 137182^{T}	Italy	Rock	NR_144975	MH878597	-
Neodevriesia queenslandica	CBS 129527^{T}	Australia	*Scaevola taccada*, leaves	JF951148	KF901839	-
Neodevriesia shakazului	CBS 133579^{T}	South Africa	*Aloe* sp., leaves	NR_111825	NG_042753	-
Neodevriesia simplex	CBS 137183^{T}	Italy	Rock	NR_155464	KF310027	-
Neodevriesia strelitziae	CBS 122379^{T}	South Africa	*Strelitzia nicolai*, leaves	MH863206	EU436763	-
Neodevriesia xanthorrhoeae	CBS 128219^{T}	Australia	*Xanthorrhoea australis*, leaves	NR_144962	HQ599606	-
Paradevriesia americana	CBS 117726^{T}	USA	Air	NR_159866	NG_059077	-
Paradevriesia compacta	CBS 118294^{T}	Spain	Limestone rock, surface	NR_144955	NG_059089	-
Paradevriesia pseudoamericana	CBS 126270^{T}	Germany	*Malus domestica*, fruit surface	NR_171743	NG_064229	-
Phaeocryptopus nudus	CBS 268.37	Germany	*Abies balsamea*	EU700371	GU301856	-
Phialophora ellipsoidea	CBS 286.47^{T}	Brazil	Human, mycetoma hand	-	AF050282	
Pseudoteratosphaeria africana	CBS 144595^{T}	Angola	Leaf spot on unidentified host	NR_163380	MK442558	-
Pseudoteratosphaeria flexuosa	CBS 111012^{T}	Colombia	*Eucalyptus globulus*	-	NG_069169	-
Rhinocladiella fasciculata	CBS 132.86^{T}	India	Decayed wood	-	NG_057784	-
Rhizosphaera kalkhofii	ATCC 26605	USA	*Picea pungens*, blue spruce needles	-	EF114706	-
Rhizosphaera kalkhofii	ATCC 46388	France	*Picea glauca* needles	AY183366	-	-
Rhizosphaera macrospora	CBS 208.79^{T}	France	*Abies alba*, needle on dead twig	NR_166003	NG_064115	
Rhizosphaera macrospora	CBS 467.82	Switzerland	*Abies alba*, endophyte	EU700368	-	-
Rhizosphaera oudemansii	CBS 427.82	Switzerland	*Abies alba*, endophyte	EU700367	-	-
Rhizosphaera oudemansii	CBS 226.83	Spain	*Abies pinsapo*, needle	EU700366	-	-
Rhizosphaera pini	CBS 206.79	France	*Abies alba*, needle on dead twig	EU700370	-	-

Rhizosphaera pseudotsugae	CBS 101222	Unknown	*Pseudotsuga menziesii*, needles	EU700369	-	-
Roesleria subterranea	CBS 320.33	Netherlands	*Malus sylvestris*, root	-	MH866908	-
Scleroconidioma sphagnicola	UAMH 9731	Canada	*Sphagnum fuscum*, lesions on thalli	NR_121294	-	-
Stylodothis puccinioides	CBS 193.58	Switzerland	*Viburnum lantana*	KY929139	-	-
Thyrinula eucalypti	CBS 145894^{T}	Australia	*Eucalyptus cloeziana*, leaves	-	HM535600	MN162605
Thyrinula eucalyptina	CPC 13748	Australia	*Eucalyptus globulus*	-	MN162255	MN162608
Xenodevriesia strelitziicola	CBS 122480^{T}	South Africa	*Strelitzia* sp.	-	MH874744	-
Xenomeris abietis	CBS 799.72	Canada	*Pseudotsuga menziesii*	-	MH872299	-

^{T}, type strain. ATCC, American Type Culture Collection (Manassas, VA, USA). CAP, Culture Collection of Alan Phillips, housed at the Lab Bugworkers | M&B-BioISI | Tec Labs – Innovation Centre, University of Lisbon (Lisbon, Portugal). CBS, CBS-KNAW Westerdijk Fungal Biodiversity Institute (Utrecht, Netherlands). CGMCC, China General Microbiological Culture Collection Center (Beijin, China). CPC, working collection of Pedro Crous, housed at the Westerdijk Fungal Biodiversity Institute (Utrecht, Netherlands). MFLUCC, Mae Fah Luang Culture Collection (Chiang Rai, Thailand). UAMH, University of Alberta Mold Herbarium and Culture Collection (Edmonton, Canada)

Table S3: Carbon source assimilation.

TEST / STRAIN	CARBON SOURCE ASSIMILATION																																															
	GLY (Glycerol)	ERY (Erythritol)	DARA (D-arabinose)	LARA (L-arabinose)	RIB (D-ribose)	DXYL (D-xylose)	LXYL (L-xylose)	ADO (D-adonitol)	MDX (methyl-ß-Dxylopyranoside)	GAL (D-galactose)	GLU (D-glucose)	FRU (D-fructose)	MNE (D-mannose)	SBE (L-sorbose)	RHA (L-rhamnose)	DUL (Dulcitol)	INO (Inositol)	MAN (D-mannitol)	SOR (D-sorbitol)	MDM (methyl-Dmannopyranoside)	MDG (methyl-Dglucopyranoside)	NAG (N-acetylglucosamine)	AMY (Amygdalin)	ARB (Arbutin)	ESC (Esculin)	SAL (Salicin)	CEL (D-cellobiose)	MAL (D-maltose)	LAC (D-lactose)	MEL (D-melibiose)	SAC (D-saccharose)	TRE (D-trehalose)	INU (Inulin)	MLZ (D-melezitose)	RAF (D-raffinose)	AMD (Amidon)	GLYG (Glycogen)	XLT (Xylitol)	GEN (Gentiobiose)	TUR (D-turanose)	LYX (D-lyxose)	TAG (D-tagatose)	DFUC (D-fucose)	LFUC (L-fucose)	DARL (D-arabitol)	LARL (L-arabitol)	GNT (potassiumgluconate)	2KG (potassium-2-keto-gluconate)	5KG (potassium-5-keto-gluconate)
A. microstrictum FMR 19067	+	+	-	+	+	+	w	-	-	-	w	w	-	w	+	-	+	+	+	-	+	+	+	w	+	+	+	+	+	+	-	-	+	+	+	w	+	+	+	+	-	+	-	-	w	w	w	-	w
A. pullulans FMR 18808	-	+	-	+	-	+	w	-	+	+	+	+	w	-	+	-	+	+	+	+	w	+	+	+	+	+	+	+	-	w	+	+	-	+	+	+	+	+	+	+	+	-	-	-	+	+	+	-	+
C. pleiosporus FMR 18827	w	w	+	+	+	+	-	-	-	-	+	+	+	w	w	w	w	+	+	-	w	w	w	-	+	-	+	+	+	+	+	+	w	+	-	w	w	w	+	+	-	w	-	w	w	w	+	-	-
E. caementiphila FMR 18977	+	-	-	-	-	w	-	w	-	w	+	+	+	+	-	-	w	+	w	-	-	-	+	+	-	w	w	-	w	-	-	w	-	-	-	-	w	w	w	+	-	+	-	-	+	+	+	-	+
E. multiformis FMR 18809	+	-	-	w	+	+	+	+	-	-	+	+	+	+	+	+	-	+	+	w	+	+	+	+	-	+	+	+	+	-	+	+	+	+	+	+	+	+	+	+	+	+	-	+	+	+	+	-	+
E. xenobiotica FMR 18810	+	-	-	w	-	+	-	w	w	+	+	+	+	+	-	-	w	-	-	+	+	-	-	-	-	-	w	w	w	+	+	+	-	-	-	-	+	-	+	+	+	w	+	w	+	-	w	-	-
E. xenobiotica FMR 19066	+	+	+	-	+	-	-	-	+	+	+	+	+	-	-	-	-	-	-	-	-	w	w	+	+	+	+	w	-	w	+	+	-	-	+	+	+	+	+	w	+	w	w	-	w	-	-	-	-
E. xenobiotica CBS 118157	+	+	+	-	+	-	-	-	+	+	+	+	+	-	-	-	-	-	-	-	-	w	w	+	+	+	+	w	-	w	+	+	-	-	+	+	+	+	+	w	+	w	w	-	w	-	-	-	-

K. epidermidis FMR 18978	-	+	w	+	w	-	+	+	+	-	+	+	+	-	-	-	w	-	-	-	+	-	-	+	+	-	+	-	-	-	w	-	-	w	w	+	+	-	+	-	-	-	-	-	-	w	+	+	+
K. perfecta FMR 18715	+	-	-	+	-	+	-	w	-	w	-	-	+	+	-	-	-	+	+	-	w	-	-	-	-	w	+	-	-	-	-	-	-	+	w	-	-	+	-	-	-	-	-	-	-	-	-	-	-
P. kinklidomatophilus CBS 147696	+	-	-	+	-	+	-	+	-	-	w	w	w	-	-	-	-	+	+	-	-	-	-	-	-	-	w	+	-	+	+	+	-	w	+	w	+	-	w	+	-	-	-	-	-	-	-	-	-

+, growth. w, slow or low growth. -, no-growth.

Table S4: Nitrogen source assimilation, osmotolerance, cycloheximide resistance, urease and DNAse production, acid from glucose and sugar fermentation for the fungal strains tested.

TEST	NITROGEN SOURCE ASSIMILATION											GLUCOSE ASSIMILATION										OSMOTOLE-RANCE		CICLOHEXI-MIDE TOLERANCE						CARBON SOURCE FERMENTATION								
**STRAIN**	KNO_3	$NaNO_2$	$(NH4)_2SO_4$	L-Lysine	Creatinine	Creatine	L-Tryptophan	L-Proline	L-Leucine	L-Ornitine	Arginine	0.1%	0.25%	0.5%	1.0%	2.0%	3.0%	4.0%	5.0%	7.5%	10%	50%	60%	0.01%	0.05%	0.1%	**UREASE**	**DNAse**	**ACID PRODUCTION**	Glucose	Galactose	Sacarose	Maltose	Lactose	Raffinose	Trehalose	Manose	Inulin
A. microstrictum FMR 19067	w	-	+	+	+	w	+	+	+	+	+	w	+	+	+	+	+	+	+	+	+	+	+	-	-	-	+	-	+	+	w	+	+	w	+	+	+	+
A. pullulans FMR 18808	+	+	+	+	-	w	+	+	+	+	+	w	+	+	+	+	+	+	+	+	+	+	w	-	-	-	+	-	+	+	+	+	+	w	+	+	+	+
C. pleiosporus FMR 18827	-	-	-	-	w	-	w	+	w	-	w	+	+	+	+	+	+	+	+	+	+	w	w	-	-	-	-	-	+	+	-	+	+	-	+	w	w	-
E. caementiphila FMR 18977	-	-	w	-	-	+	+	+	+	+	+	w	w	w	+	+	+	+	+	w	+	-	-	-	-	-	+	-	-	+	+	w	w	+	w	+	w	w
E. multiformis FMR 18809	+	+	+	+	+	+	+	+	+	+	+	+	+	+	+	+	+	+	+	+	+	-	-	-	-	-	+	-	-	+	w	w	+	-	w	+	+	w
E. xenobiotica FMR 18810	+	+	+	+	+	+	+	+	+	+	+	+	+	+	+	+	+	+	+	+	+	w	-	-	-	-	+	-	-	+	+	+	+	+	w	+	+	w
E. xenobiotica FMR 19066	+	+	+	+	+	+	+	+	+	+	+	+	+	+	+	+	+	+	+	+	+	+	w	+	+	+	+	-	-	+	w	+	+	+	+	+	+	w
E. xenobiotica CBS 118157	+	+	+	+	+	+	+	+	+	+	+	+	+	+	+	+	+	+	+	+	+	+	w	+	+	+	+	-	-	+	+	+	+	+	+	+	+	+
K. epidermidis FMR 18978	+	+	+	+	+	+	+	+	+	+	+	+	+	+	+	+	+	+	+	+	+	w	-	+	+	+	w	-	-	w	-	+	w	-	w	w	w	-
K. perfecta FMR 18715	-	-	-	-	-	-	-	-	-	-	-	+	+	w	w	w	w	w	w	w	w	-	-	-	-	-	-	-	-	-	-	-	-	-	-	-	-	-
P. kinklidomatophilus CBS 147696	-	-	-	-	-	-	-	-	-	-	-	+	w	w	+	+	+	+	+	+	+	-	-	-	-	-	-	-	-	-	-	-	-	-	-	-	-	-

+ = growth, w = slow or low growth, **-** = no-growth.

Table S5: Halotolerance, thermotolerance, pH tolerance, and gelatinase production for the fungal strains tested.

TEST / STRAIN	HALOTOLERANCE									THERMOTOLERANCE (14 d, mm)									pH TOLERANCE										GELATINAS
	2.5 % NaCl	5.0 % NaCl	10 % NaCl	2.5 %	5.0 %	10 %	2.5 %	5.0 %	10 % $CaCl_2$	5 °C	15 °C	18°C	25 °C	30 °C	35 °C	37 °C	40 °C	45 °C	3	4	5	6	7	8	9	10	11	12	
A. microstrictum FMR 19067	-	-	-	+	+	+	+	+	-	10 ± 0.0	58 ± 0.0	68 ± 1	61 ± 1	11 ± 1	-	-	-	-	+	+	+	+	+	+	+	+	-	-	10.5 ± 0.5
A. pullulans FMR 18808	+	+	+	+	+	+	+	w	-	15 ± 1	62 ± 0.0	75 ± 1	77.5 ± 0.5	27.5 ± 0.5	1.5 ± 0.5	-	-	-	+	+	+	+	+	+	+	+	+	-	11 ± 0.0
C. pleiosporus FMR 18827	w	-	-	+	+	w	w	w	-	4.75 ± 0.25	32.5 ± 1.5	39 ± 1	35.5 ± 0.5	1.5 ± 0.5	-	-	-	-	+	+	+	+	+	+	+	-	-	-	5.5 ± 0.5
E. caementiphila FMR 18977	-	-	-	+	+	+	w	-	-	1.25 ± 0.25	2 ± 0.0	2.5 ± 0.5	3.5 ± 0.5	-	-	-	-	-	+	+	+	+	+	+	+	-	-	-	-
E. multiformis FMR 18809	+	w	-	+	+	+	+	+	-	1.5 ± 0.0	5 ± 0.0	6 ± 0.0	11 ± 1	6.5 ± 0.5	-	-	-	-	+	+	+	+	+	+	+	+	+	-	-
E. xenobiotica FMR 18810	+	w	-	+	+	+	+	+	-	3 ± 1	6.5 ± 0.5	8.5 ± 0.5	11 ± 1	5 ± 0.0	1.25 ± 0.25	-	-	-	+	+	+	+	+	+	+	+	+	-	-
E. xenobiotica FMR 19066	+	+	-	+	+	+	+	+	-	-	6 ± 0.0	7 ± 1	13 ± 0.0	14 ± 1	5 ± 1	-	-	-	+	+	+	+	+	+	+	+	+	+	-

E. xenobiotica CBS 118157	+	+	-	+	+	+	+	+	-	4 ± 0.0	7.5 ± 0.5	9.5 ± 0.5	15.5 ± 0.5	10 ± 1	-	-	-	-	+	+	+	+	+	+	+	+	+	-	-
K. epidermidis FMR 18978	+	-	-	+	+	+	+	+	-	-	6 ± 0.0	9.5 ± 0.5	14.5 ± 0.5	10.5 ± 0.5	-	-	-	-	w	+	+	+	+	+	+	+	+	-	-
K. perfecta FMR 18715	-	-	-	+	w	-	-	-	-	-	3.5 ± 0.5	3 ± 0.0	2 ± 0.0	-	-	-	-	-	-	+	+	+	+	+	+	+	+	+	-
P. kinklidomatophilus CBS 147696	-	-	-	w	-	-	-	-	-	2.5 ± 0.5	3 ± 0.0	2.5 ± 0.5	2.75 ± 0.25	-	-	-	-	-	+	+	+	w	-	-	-	-	-	-	9.5 ± 0.5

+ = growth, w = slow or low growth, - = no-growth, mm = millimeters.
